# Mechanism of orphan subunit recognition during assembly quality control

**DOI:** 10.1016/j.cell.2023.06.016

**Published:** 2023-08-03

**Authors:** Yuichi Yagita, Eszter Zavodszky, Sew-Yeu Peak-Chew, Ramanujan S. Hegde

**Affiliations:** 1 MRC Laboratory of Molecular Biology, Cambridge CB2 0QH, UK

**Keywords:** protein quality control, degradation, protein homeostasis, ubiquitination, E3 ligase, chaperonin

## Abstract

Cells contain numerous abundant molecular machines assembled from multiple subunits. Imbalances in subunit production and failed assembly generate orphan subunits that are eliminated by poorly defined pathways. Here, we determined how orphan subunits of the cytosolic chaperonin CCT are recognized. Several unassembled CCT subunits recruited the E3 ubiquitin ligase HERC2 using ZNRD2 as an adaptor. Both factors were necessary for orphan CCT subunit degradation in cells, sufficient for CCT subunit ubiquitination with purified factors, and necessary for optimal cell fitness. Domain mapping and structure prediction defined the molecular features of a minimal HERC2-ZNRD2-CCT module. The structural model, whose key elements were validated in cells using point mutants, shows why ZNRD2 selectively recognizes multiple orphaned CCT subunits without engaging assembled CCT. Our findings reveal how failures during CCT assembly are monitored and provide a paradigm for the molecular recognition of orphan subunits, the largest source of quality control substrates in cells.

## Highlights

• The E3 ligase HERC2 triggers degradation of unassembled chaperonin subunits
• ZNRD2 is a chaperonin-specific adaptor that is essential for HERC2 recruitment
• Folded rather than misfolded chaperonin subunits are targets for ZNRD2
• Structural predictions and mutagenesis reveal mechanism of target recognition

## Graphical abstract

**Figure:**
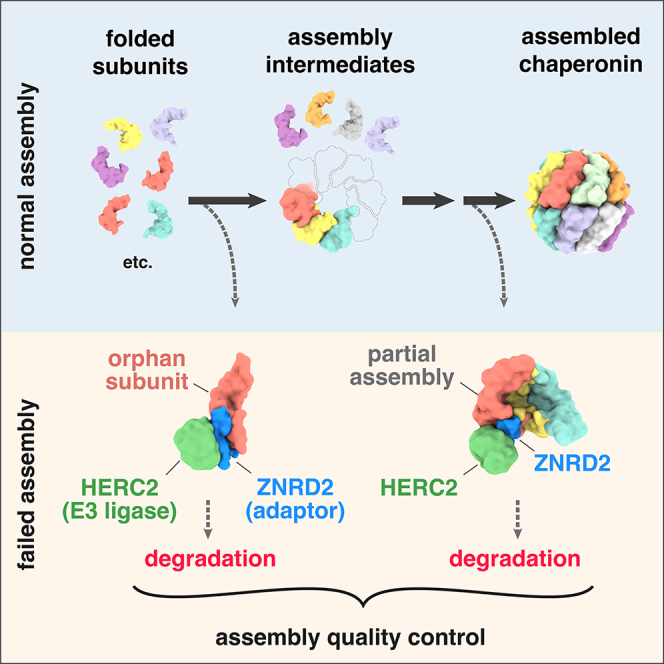

## Teaser

Biochemical and structural analyses reveal how cells recognize and degrade folded but unassembled subunits arising from inefficient or failed assembly of a multi-protein complex.

## Introduction

Many of the cell’s most abundant proteins are subunits of larger complexes, such as ribosomes, proteasomes, chaperonin, tubulin, hemoglobin, and others. Most of these complexes are heteromeric structures comprising two or more different constituents. This poses two problems for the cell. First, the individual constituents must be produced at their desired final stoichiometry and correctly assembled with each other.^1^^,^^2^ Second, any unassembled or incompletely assembled products (hereafter termed orphans) must be identified and eliminated.^3^^,^^4^^,^^5^ How the assembly pipeline is monitored for failures by cellular quality control (QC) is poorly understood for the majority of protein complexes.

The existence of QC for orphans has been appreciated for decades.^6^ Not only are heterologously expressed subunits typically unstable^7^^,^^8^ but also elimination of one subunit usually destabilizes its partners.^9^^,^^10^^,^^11^^,^^12^ These experimental perturbations resemble exaggerated versions of imbalances that occur in normal cells due to the inherent noisiness of gene expression. Consistent with this idea, pulse-chase analyses show that subunits of multi-protein complexes often display bi-phasic degradation.^13^^,^^14^ The rapid phase presumably corresponds to orphan degradation, while the slow phase reflects the population of subunits that assembled successfully into a long-lived complex. These analyses indicate that protein complex assembly is the largest contributor to protein biogenesis failures, highlighting the centrality of orphan QC for maintaining proteostasis in cells.

Indeed, acutely amplifying subunit imbalances by inducing aneuploidy incurs severe fitness costs, protein aggregation, and proteostasis collapse.^15^^,^^16^^,^^17^^,^^18^ Even imbalances in a single abundant protein can have severe consequences. For example, reduced production of the β-globin subunit in β-thalassemias leads to orphaned α-globin subunits whose accumulation causes protein aggregation, oxidative stress, membrane damage, and cell death.^19^ Yet, despite these fitness costs, aneuploidies are frequent in cancer cells,^20^ implying the existence of mitigating mechanisms for orphan degradation.^21^ These orphan QC systems are only starting to be defined, with even less insight into the mechanisms underlying the decisive step of substrate recognition.^4^^,^^5^ These pathways are not only central to the maintenance of proteostasis but are also potential vulnerabilities in aneuploid cancers.

Three of the most abundant and complicated multi-subunit complexes are the ribosome, proteasome, and chaperonin.^22^ The assembly of the first two has been studied in some detail for many years,^23^^,^^24^ with QC factors only recently emerging. Orphan ribosomal proteins in the nucleus are ubiquitinated by Tom1 (HUWE1 in mammals),^25^^,^^26^ whereas orphan cytosolic ribosomal proteins are ubiquitinated by UBE2O.^27^^,^^28^ Reconstitution experiments suggest that in both cases, the E3 ligase recognizes the orphan directly via regions of polypeptide that would be buried in the assembled ribosome. By contrast, orphan PSMC5, a subunit of the proteasome, is recognized indirectly by the E3 ligase HERC1 via PAAF1, a proteasome assembly factor.^14^ The molecular details of recognition are not understood for any of these QC factors.

In contrast to ribosomes and proteasomes, almost nothing is known about the assembly and QC of the comparably abundant eukaryotic chaperonin subunits. Known as CCT or TRiC, cytosolic chaperonin is a cylindrical ∼1 MDa complex comprising two rings.^29^ Each ring contains eight homologous ATPase subunits (CCT1 through CCT8) arranged in a precise order. This structure poses a challenging assembly problem that could be prone to failure. Consistent with this idea, proteomic analyses find that a subpopulation of several CCT subunits is rapidly degraded shortly after synthesis, indicative of constant CCT orphan generation.^13^^,^^14^ Neither the factors that mediate degradation of orphan CCTs nor the mechanism of their recognition are known. The exceptional abundance of CCT,^22^ its central role in biology,^30^ and the emerging importance of orphan QC for cellular proteostasis^3^^,^^4^^,^^5^ motivated us to investigate this problem.

## Results

### Candidate QC factors for orphan chaperonin subunits

**Figure S1:**
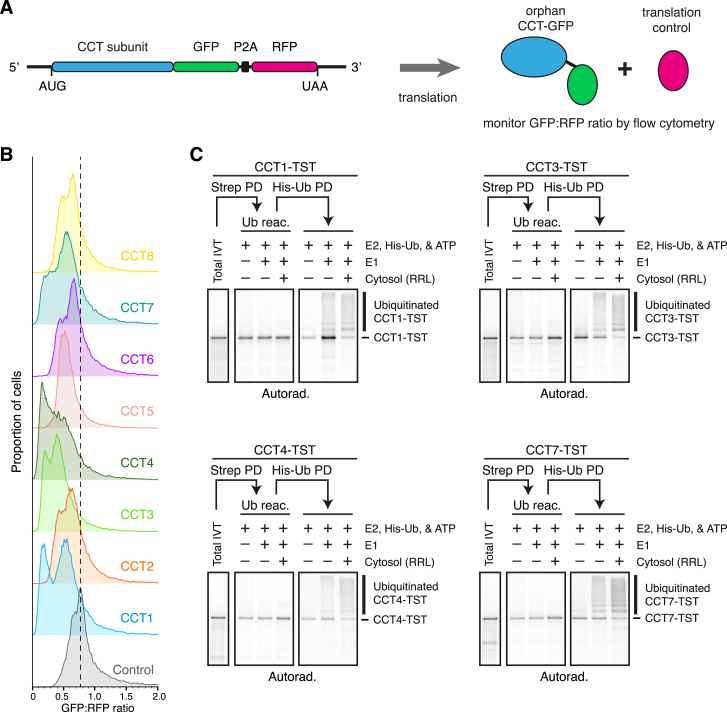
Degradation and ubiquitination of unassembled chaperonin subunits, related to Figure 1 (A) Diagram illustrating the mRNA coding for the dual-color CCT reporter construct and expected protein products. GFP-fused CCT subunit and RFP are produced from the same mRNA through ribosomal skipping at a viral P2A sequence. The GFP:RFP fluorescence ratio reports the stability of a test GFP-fusion relative to the internal RFP control. (B) HEK293T cells were transfected with control or CCT reporter constructs and analyzed by flow cytometry. The control construct does not contain a CCT subunit and only has GFP and RFP. The dotted line indicates the peak from the histogram of this control construct. Note that each of the CCT subunit fusions to GFP is less stable than GFP alone, with CCT4 being among the least stable and CCT8 being among the most stable. (C) ^35^S-methionine-labeled CCT subunits with a C-terminal TST were translated in RRL (total IVT) and affinity-purified via TST under native conditions. The affinity-purified products were split into three equal aliquots, incubated with E1, E2 (UBCH5), His-Ub, ATP, and RRL, as indicated. The products of this post-purification ubiquitination reaction were either analyzed directly (Ub reac.) or after His-Ub pull-down under denaturing conditions (His-Ub PD). ^35^S-methionine-labeled CCT subunits were visualized by autoradiography.

**Figure 1:**
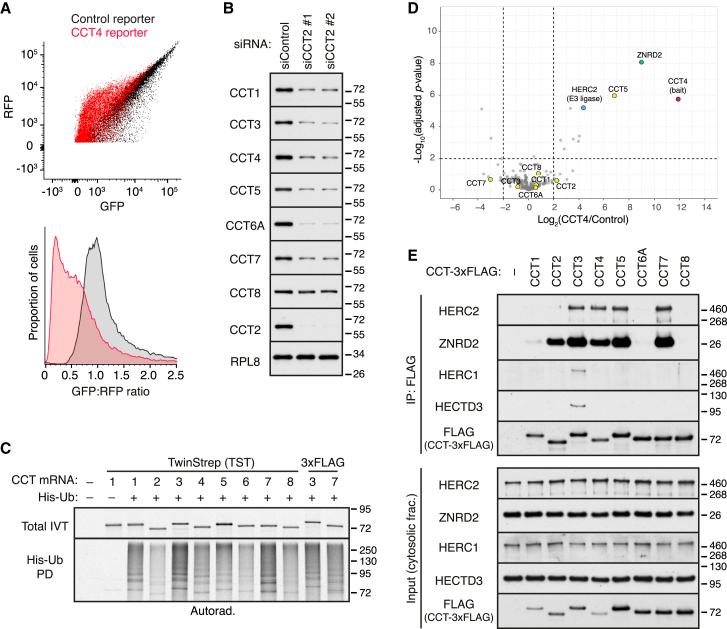
Interactors of unassembled chaperonin subunits destined for degradation (A) HEK293T cells were transfected with a GFP-RFP dual-color reporter of orphan CCT4 degradation (red; see Figure S1A) or a control GFP-RFP reporter lacking the CCT4 insert (black), then analyzed by flow cytometry. CCT4 is fused to GFP, with RFP serving as a translation control. Shown are overlaid scatter plots of individual transfected cells (top) and the corresponding histograms of the GFP:RFP ratio (bottom). (B) Cells were treated with control or CCT2-targeting small interfering RNAs (siRNAs) for 72 h, and total cell lysates were analyzed by immunoblotting for the proteins indicated on the left. (C) CCT subunits containing a C-terminal TwinStrep tag (TST) or 3xFLAG tag were translated in rabbit reticulocyte lysate (RRL) containing ^35^S-methionine and His-tagged ubiquitin (His-Ub). Samples were analyzed directly (total IVT, *in vitro* translation) or after ubiquitin pull-down under denaturing conditions via the His-tag (His-Ub PD). (D) CCT4-TST was translated in RRL, affinity-purified under native conditions, and analyzed by label-free quantitative mass spectrometry. Proteins in the upper right quadrant are significantly enriched with CCT4. (E) Cells transiently expressing 3xFLAG-tagged CCT subunits were subjected to anti-FLAG immunoprecipitation (IP) under non-denaturing conditions. Input and IP samples were analyzed by immunoblotting for the indicated proteins. See also Figures S1 and S2.

Fluorescent reporters of each orphan CCT subunit were generated by fusing GFP to the C terminus (Figure S1A), which is likely to impede assembly into a complete CCT in which the C terminus is normally buried inside the double-ring structure. Relative to a co-linear RFP control, several CCT-GFP subunits were degraded to varying degrees upon overexpression in cultured cells (Figures 1A and S1B). Similarly, knockdown of CCT2 results in degradation of most other endogenous CCT subunits to varying degrees (Figure 1B), as observed in earlier studies.^9^ Some endogenous orphaned CCT subunits (e.g., CCT5 and CCT6A) are degraded more effectively than their corresponding GFP-tagged reporters (compare Figure 1B with Figure S1B). This observation indicated that CCT1, 3, 4, and 7 are most likely to be reliable GFP reporters, whereas others are less useful due to a stabilizing effect of the GFP tag. Thus, several exogenous and endogenous orphan CCTs are degraded in cells, indicating the existence of one or more QC processes for their elimination.

**Figure S2:**
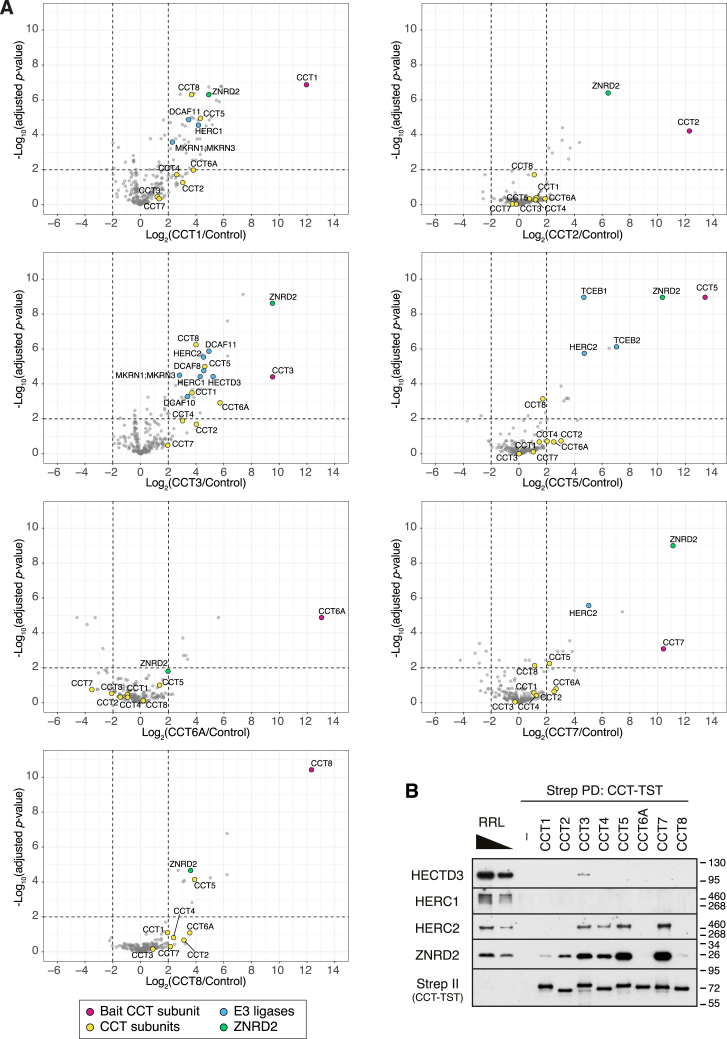
Identification of candidate quality control factors for unassembled CCT subunits, related to Figure 1 (A) CCT subunits with a C-terminal TST were translated in RRL, affinity-purified under native conditions via the TST, and analyzed by label-free quantitative mass spectrometry. The volcano plots illustrate proteins enriched in CCT subunit pull-downs compared with control pull-downs (see method details for details). The log_2_ fold difference (x axis) is plotted against the −log_10_ adjusted p value (y axis). The statistical analysis was done using a two-sided Student’s t test with Benjamini-Hochberg correction for multiple comparisons. The bait and non-bait CCT subunits are shown as magenta and yellow dots, respectively. E3 ligases, including components of E3 ligase complexes, are in blue. ZNRD2 (also called SSSCA1) is in green. (B) CCT subunits with a C-terminal TST were translated in RRL, affinity-purified under native conditions, and analyzed by immunoblotting for HECTD3, HERC1, HERC2, and ZNRD2. The CCT subunit was detected via the TST. Two amounts of total RRL were analyzed in parallel.

Translation in reticulocyte lysate of individual CCT subunits without their partners led to their ubiquitination (Figure 1C), suggesting that QC of orphan CCT subunits may be active in this system. Native affinity purification of *in vitro*-translated CCT subunits co-purified an associated E3 ubiquitin ligase activity, as evidenced by subsequent ubiquitination upon addition of E1 and E2 enzymes, ubiquitin, and ATP (Figure S1C). Candidates for the responsible ligase were identified by mass spectrometry of larger-scale purifications (Figures 1D and S2A). The major enriched product in each case was the translated CCT subunit, which is orphaned as indicated by the relative paucity of most other CCT subunits. Five of the orphaned CCT subunits co-purified ubiquitin ligase(s), of which HERC2 was seen with CCT3, CCT4, CCT5, and CCT7 (Figures 1D, S2A, and S2B). The little-studied protein ZNRD2 (also called SSSCA1) was strongly enriched with nearly every CCT subunit, particularly those that bind HERC2, an observation we will come back to later.

The major interactions observed *in vitro* using reticulocyte lysate were recapitulated in cultured cells using co-immunoprecipitation (co-IP) of overexpressed (and hence, mostly orphaned) FLAG-tagged CCT subunits (Figure 1E). Notably, CCT3 displayed interactions with several ubiquitin ligases (including HERC2), perhaps indicating multiple redundant degradation pathways. At the other extreme, CCT8 did not engage any obvious QC factors (Figure S2A) and was a poor substrate for degradation in reporter assays or after knockdown of other CCTs (Figures 1B and S1B). These results suggest that QC of orphan CCT subunits is not uniform, perhaps reflecting their differential needs based on how the assembly pathway proceeds. Among the QC candidates, HERC2 was particularly attractive for further investigation because it seems to engage multiple CCTs *in vitro* and in cells.

### HERC2 is required for efficient chaperonin subunit degradation

**Figure 2:**
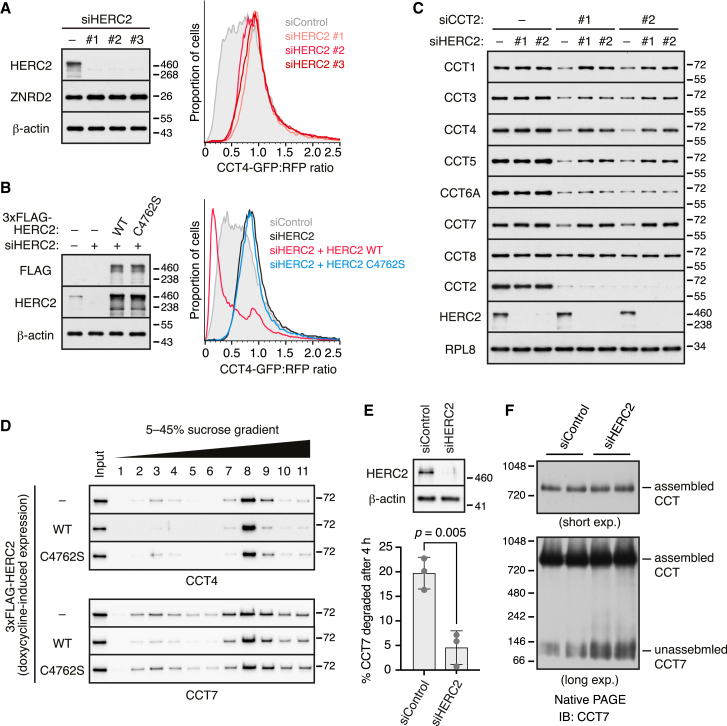
Degradation of unassembled chaperonin subunits requires the E3 ligase HERC2 (A) Cells treated with control or HERC2-targeting siRNAs were transfected with the dual-color CCT4 reporter and analyzed by immunoblotting (left) and flow cytometry (right). (B) Cells treated with the indicated siRNAs were transfected with the CCT4 reporter and siRNA-resistant 3xFLAG-HERC2 (wild type [WT]) or its catalytically inactive mutant (C4762S), as indicated. Cells were analyzed by immunoblotting (left) and flow cytometry (right). (C) Cells were transfected with CCT2-targeting siRNAs, together with or without HERC2-targeting siRNAs, as indicated. Total cell lysates were prepared at 72 h post-transfection and analyzed by immunoblotting for the indicated proteins. (D) Flp-In T-REx 293 cells stably integrated with an inducible 3xFLAG-HERC2 construct (WT), its catalytically inactive mutant (C4762S), or empty vector as a control (−) were treated with doxycycline to induce the expression of each HERC2 construct (see Figure S3E). Cytosolic extracts (input) were separated on a 5%–45% sucrose gradient and analyzed by immunoblotting for CCT4 and CCT7. Unassembled CCT subunits migrate in fractions 2–4, whereas assembled CCT migrates in fractions 7–9. (E) MCF7 cells treated with control or HERC2-targeting siRNAs were subjected to a pulse-chase experiment with ^35^S-methionine to monitor endogenous CCT7 degradation (see Figure S3G). Top panels show immunoblots of cell lysates for HERC2 and β-actin. The graph shows the proportion of radiolabeled CCT7 degraded after a 4-h chase. Data show mean ± SD, along with individual data points of 3 replicates. p = 0.005 by unpaired two-tailed t test. (F) Cytosolic extracts of MCF7 cells treated with control or HERC2-targeting siRNAs were separated by native PAGE and immunoblotted for CCT7. Two exposures of the blot are shown. See also Figure S3.

**Figure S3:**
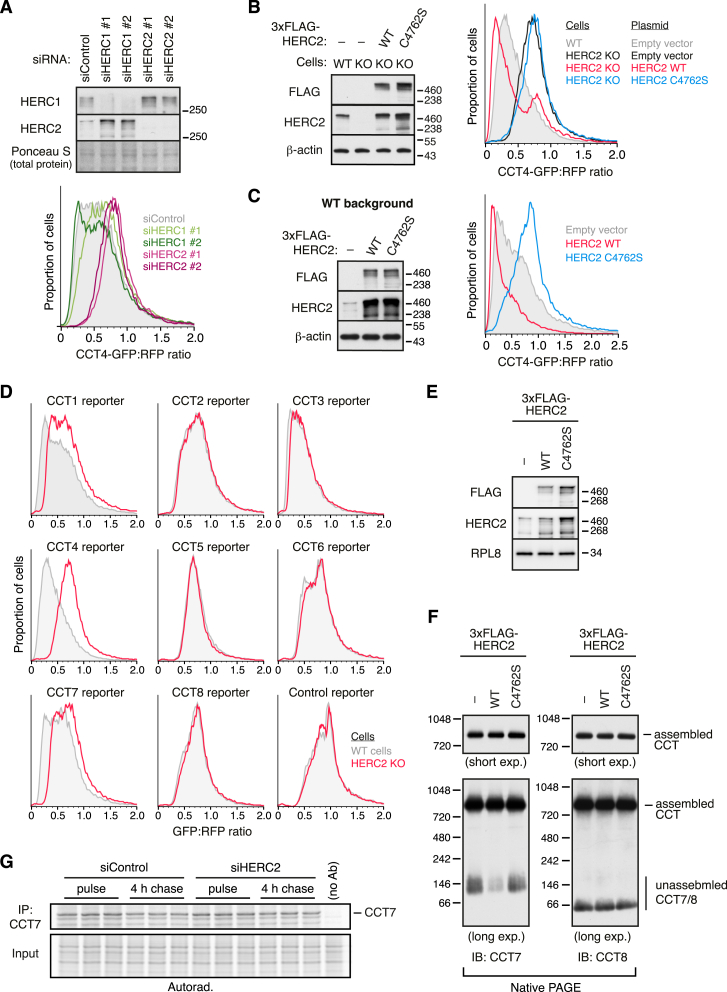
HERC2-dependent degradation of unassembled chaperonin subunits, related to Figure 2 (A) CCT4 reporter degradation (bottom) was assessed by flow cytometry in HEK293T cells knocked down for HERC1 or HERC2. Total cell lysates were also analyzed by immunoblotting to confirm depletion of the target gene products (top). (B) WT and HERC2-KO Flp-In T-REx 293 cells were transiently transfected with the CCT4 reporter along with empty vector, 3xFLAG-HERC2 (WT), or catalytically inactive HERC2 (C4762S). CCT4 reporter degradation was assessed by flow cytometry (right), and total cell lysates were analyzed by immunoblotting as indicated (left). (C) HEK293T cells were co-transfected with the CCT4 reporter and empty vector, 3xFLAG-HERC2 (WT), or catalytically inactive HERC2 (C4762S). Cells were analyzed by flow cytometry (right), and total cell lysates were analyzed by immunoblotting (left). (D) WT and HERC2-KO Flp-In T-REx 293 cells were transiently transfected with the indicated reporter constructs and analyzed by flow cytometry. The data for CCT4 is from (B) and is shown again for the purpose of comparison to the other CCT subunits. (E and F) Flp-In T-REx 293 cells stably integrated with an inducible 3xFLAG-HERC2 construct (WT), its catalytically inactive mutant (C4762S), or empty vector as a negative control (−) were treated with doxycycline to induce the expression of each HERC2 construct. Cytosolic extracts were separated by SDS-PAGE (E) or native PAGE (F) and analyzed by immunoblotting as indicated. In (F), two exposures of the blots are shown for both CCT7 and CCT8. (G) MCF7 cells treated with either control or HERC2-targeting siRNAs were radiolabeled with ^35^S-methionine for 30 min and chased for 4 h. The pulse-labeled cells and pulse-chased cells were lysed under denaturing conditions and analyzed directly (input) or subjected to IP using anti-CCT7 antibody. As a specificity control, a pulse-labeled sample was subjected to IP without antibody (no Ab). Input and IP samples were separated by SDS-PAGE and visualized by autoradiography.

We focused on CCT4 because it co-purified with only one prominent candidate E3 ligase (Figure 1D) and because its fluorescent reporter faithfully recapitulated the efficient degradation seen with endogenous orphaned CCT4 (Figures 1A and 1B). We found that knockdown of HERC2 stabilized a fluorescent reporter for orphan CCT4 (Figure 2A). Knockdown of HERC1, a related ubiquitin ligase involved in QC during proteasome assembly,^14^ had little or no effect on the CCT4 reporter (Figure S3A). Similar results were seen in HERC2 knockout cells generated by CRISPR (Figure S3B). In both knockdown and knockout cells, re-expression of HERC2 restored CCT4 degradation, leading to even lower levels of CCT4 reporter than wild-type (WT) cells due to HERC2 overexpression (Figures 2B and S3B). HERC2 with a mutated catalytic cysteine (C4762S) in its HECT domain failed to rescue CCT4 degradation in HERC2 knockdown or knockout cells. In WT cells, overexpressed HERC2 triggered even more degradation of the CCT4 reporter (Figure S3C). By contrast, HERC2(C4762S) stabilized the CCT4 reporter, consistent with a dominant-negative effect (Figure S3C).

HERC2 was also required for degradation of endogenous orphaned CCT4. In these experiments, we found that CCT4 degradation after knockdown of CCT2 was blunted in HERC2 knockdown cells (Figure 2C). The level of stabilization was almost complete, indicating that for this subunit, HERC2 is the major pathway of degradation. Several other CCT subunits that are also degraded after CCT2 knockdown were at least partially stabilized by HERC2 knockdown (Figure 2C). Of these, CCT1 (also called TCP1) and CCT7 were most similar to CCT4 in being strongly dependent on HERC2. Consistent with this conclusion, loss of HERC2 also stabilized fluorescent orphan CCT1 and CCT7 reporters (Figure S3D). Although this was expected for CCT7, the stabilization was unexpected for CCT1. As described later, the failure to recover appreciable HERC2 with CCT1 in the initial co-IP experiments seems to be due to loss of HERC2 during the IP and not a lack of interaction.

CCT3 degradation was only slightly dependent on HERC2 (Figure 2C), indicating the existence of other degradation pathways. As expected from this result, the fluorescent CCT3 orphan reporter was not noticeably impacted by loss of HERC2 (Figure S3D). The several other E3 ligases that were found to interact with CCT3 are attractive candidates for its QC (Figure S2A), but this was not explored further here. CCT6A was not HERC2-dependent (Figures 2C and S3D), as expected from its absence of interaction with HERC2 (Figures 1E and S2). Finally, CCT8 was not degraded after CCT2 knockdown (Figures 1B and 2C), indicating that it is not recognized as a QC substrate in these cells. Collectively, these findings indicate that when CCT subunits are orphaned by either overexpression or knockdown of CCT2, several of them are degraded by a HERC2-dependent pathway.

Earlier studies have shown that even under normal conditions, subunits of many protein complexes, including CCT, are partially orphaned due to imbalanced synthesis.^13^^,^^14^ Consistent with these observations, size fractionation of cell lysates by centrifugation through a sucrose gradient or separation by native gel electrophoresis revealed a minor population (∼5%) of unassembled CCT4, CCT7, and CCT8 (Figures 2D, S3E, and S3F). This is not due to post-lysis CCT disassembly because overexpression of HERC2, but not the inactive HERC2(C4762S), selectively diminished the unassembled population of CCT4 and CCT7 (Figures 2D and S3F), but not CCT8 (Figure S3F), with which HERC2 does not associate (Figures 1E and S2). Thus, a sizable population of multiple CCT subunits are orphaned even under normal conditions, and the degradation of a subset of these can be stimulated by HERC2.

Consistent with this conclusion, we found in radiolabeled pulse-chase experiments that ∼20% of newly made CCT7 is degraded during a 4-h chase (Figures 2E and S3G). This degradation was almost completely eliminated in cells knocked down for HERC2. As expected from this result, native gels showed that the steady-state level of unassembled endogenous CCT7 is selectively stabilized in HERC2 knockdown cells (Figure 2F). Thus, just as for CCT7 artificially orphaned by knockdown of CCT2, the minor population of CCT7 that is normally orphaned due to imbalanced expression or failed assembly is also degraded by a pathway dependent on the interacting protein HERC2.

### ZNRD2 is an adaptor between HERC2 and chaperonin subunits

**Figure 3:**
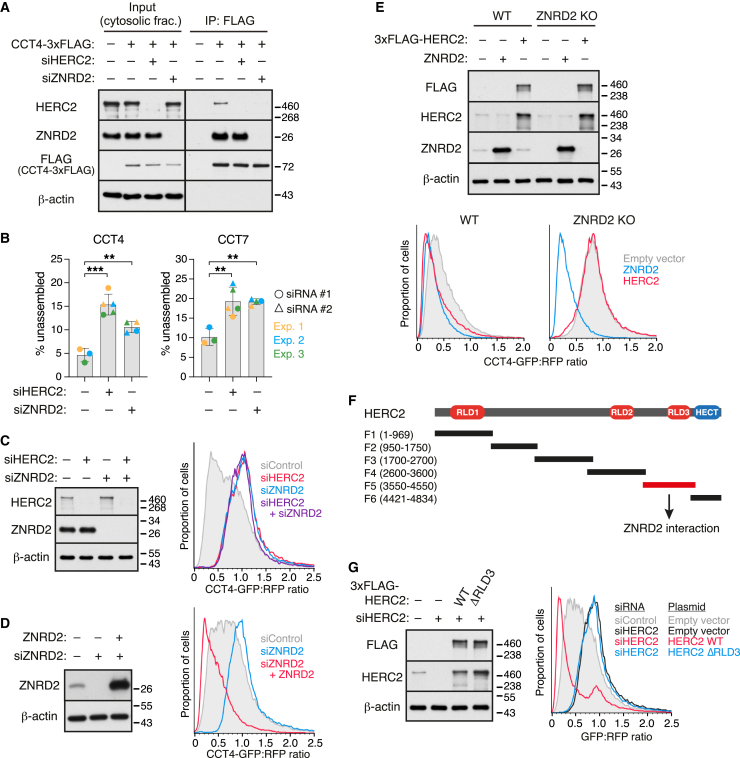
HERC2 recognizes chaperonin subunits using the adaptor ZNRD2 (A) Cells treated with siRNAs against HERC2 or ZNRD2 were transfected with CCT4-3xFLAG, followed by non-denaturing IP using anti-FLAG affinity resin. Input and IP samples were analyzed by immunoblotting for the indicated proteins. (B) Cytosolic extracts from cells treated with siRNAs targeting HERC2, ZNRD2, or control were fractionated by sucrose gradient centrifugation to quantify the proportion of unassembled CCT4 and CCT7 subunits (see Figures S4A and S4B). Data are mean ± SD of data points (dots) from three independent experiments (color-coded). ^∗∗^p < 0.01 and ^∗∗∗^p < 0.001 vs. control siRNA by one-way ANOVA with Dunn’s multiple comparisons test. (C) Cells treated with the indicated siRNAs were transfected with the dual-color CCT4 reporter and analyzed by immunoblotting (left) and flow cytometry (right). (D) Cells treated with siRNAs against ZNRD2 were transfected with the CCT4 reporter and siRNA-resistant ZNRD2, followed by immunoblotting (left) and flow cytometry (right). (E) Wild-type (WT) and ZNRD2-knockout (KO) Flp-In T-REx 293 cells were transfected with the dual-color CCT4 reporter and either 3xFLAG-HERC2, ZNRD2, or empty vector. Cells were analyzed by immunoblotting (top) and flow cytometry (bottom). (F) Diagram illustrating the domain structure of human HERC2 (4,834 amino acids) and overlapping HERC2 fragments tested for interaction with ZNRD2 in Figure S4G. HERC2 contains three RCC1-like domains (RLD1–3) and a C-terminal HECT E3 ligase catalytic domain (HECT). The HERC2 fragment containing RLD3 (F5; highlighted in red) interacts with ZNRD2 (see Figure S4G). (G) Cells treated with HERC2-targeting siRNA were transfected with the CCT4 reporter together with empty vector, siRNA-resistant 3xFLAG-HERC2 (WT), or its deletion mutant lacking RLD3 (ΔRLD3). Cells were analyzed by immunoblotting (left) and flow cytometry (right). See also Figure S4.

HERC2 might recognize its targets directly or through the use of an adaptor. The similar pattern of interaction with CCT subunits for both HERC2 and ZNRD2 hinted that ZNRD2 might be such an adaptor (Figures 1E and S2B). Co-IP experiments showed that the interaction between orphan CCT4 and endogenous HERC2 was lost when ZNRD2 was knocked down (Figure 3A). By contrast, the CCT4-ZNRD2 interaction was not dependent on HERC2. Thus, ZNRD2 is essential for HERC2 interaction with orphan CCT4.

**Figure S4:**
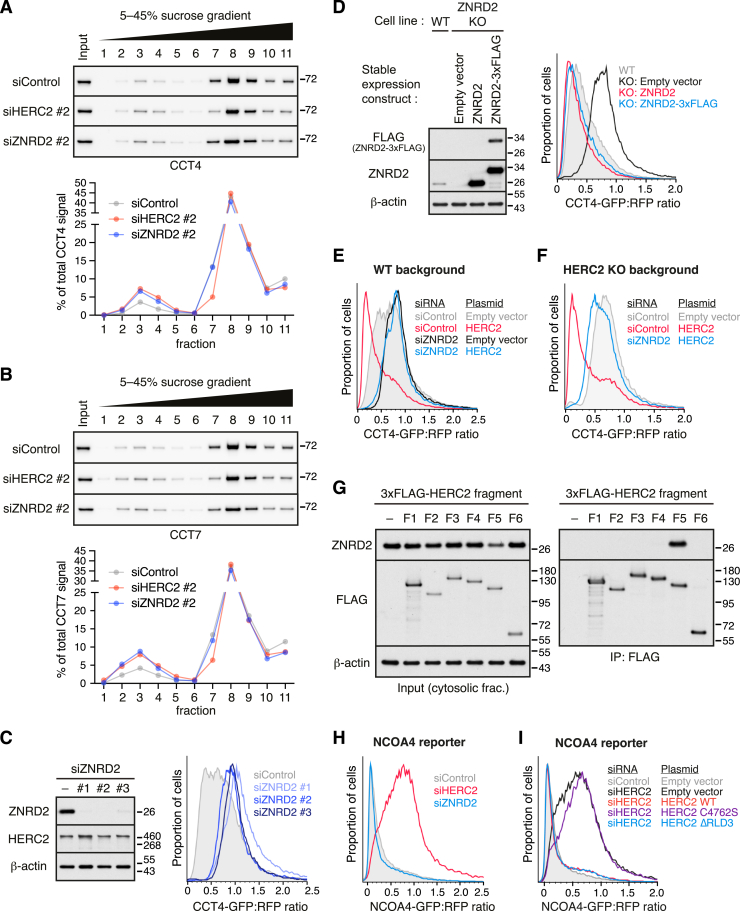
Recognition of unassembled chaperonin subunits via the adaptor ZNRD2, related to Figure 3 (A and B) Cytosolic extracts (input) from HEK293T cells treated for 5 days with the indicated siRNAs were separated into 11 fractions on a 5%–45% sucrose gradient. Input and gradient fractions were analyzed by immunoblotting for CCT4 (A) and CCT7 (B), and the signal intensities of the bands in each fraction were measured by densitometry. The graphs below the blots depict the distributions of CCT4 and CCT7 across the gradient. The proportion of unassembled CCT4 and CCT7 migrating in fractions 2–4 was used for the quantification shown in Figure 3B. Assembled CCT migrates primarily in fractions 7–9. (C) Cells treated with control or ZNRD2-targeting siRNAs were transfected with the CCT4 reporter and analyzed by immunoblotting (left) and flow cytometry (right). (D) WT Flp-In T-REx 293 cells and ZNRD2-KO cells with stably integrated empty vector, untagged ZNRD2, or ZNRD2-3xFLAG were transfected with the CCT4 reporter. Cells were analyzed by flow cytometry (right), and total cell lysates were analyzed by immunoblotting (left). (E) HEK293T cells were treated with control or ZNRD2-targeting siRNAs, transfected with the CCT4 reporter and either empty vector or 3xFLAG-HERC2, and analyzed by flow cytometry. (F) HERC2-KO Flp-In T-REx 293 cells were treated with control or ZNRD2-targeting siRNAs, transfected with the CCT4 reporter and either empty vector or 3xFLAG-HERC2, and analyzed by flow cytometry. (G) The indicated HERC2 fragments with an N-terminal 3xFLAG tag (F1–F6) were transiently expressed in HEK293T cells and immunoprecipitated under non-denaturing conditions with anti-FLAG affinity resin (IP). Input (left) and IP samples (right) were analyzed by immunoblotting. (H) A dual-color NCOA4 reporter (as in Figure S1A) was assessed for degradation by flow cytometry in HEK293T cells knocked down for HERC2 or ZNRD2. NCOA4 degradation, which is normally very efficient as indicated by the low GFP:RFP ratio, was inhibited by HERC2 knockdown but not ZNRD2 knockdown. (I) HEK293T cells treated with control or HERC2-targeting siRNAs were transfected with the NCOA4 reporter together with empty vector or the indicated 3xFLAG-HERC2 constructs. In HERC2-knockdown cells, re-expression of HERC2 WT and ΔRLD3, but not catalytically inactive C4762S mutant, restored NCOA4 degradation.

Consistent with this role, knockdown of ZNRD2 stabilized the small fraction of endogenous CCT4 and CCT7 orphans that are observed in unperturbed cells (Figures 3B, S4A, and S4B). The level of stabilization was comparable to that seen with HERC2 knockdown. ZNRD2 knockdown also stabilized the CCT4 reporter in both knockdown and knockout cells (Figures 3C, S4C, and S4D), with no further stabilization when HERC2 was also knocked down (Figure 3C). Re-expression of ZNRD2 in knockdown or knockout cells restored CCT4 reporter degradation beyond that observed even in WT cells (Figures 3D and S4D). This enhanced degradation was also seen with ZNRD2 overexpression in WT cells, as had been observed with HERC2 (Figure 3E). The ability of overexpressed HERC2 to stimulate CCT4 reporter degradation was strictly dependent on ZNRD2 (Figures 3E, S4E, and S4F).

Analysis of ZNRD2 co-IP with various fragments of HERC2 identified a region containing the third RCC1-like domain (RLD3) as the key interaction domain (Figures 3F and S4G). A deletion mutant of HERC2 lacking RLD3 (ΔRLD3) was completely inactive for orphan CCT4 degradation and could not rescue HERC2 knockdown cells (Figure 3G). Importantly, ZNRD2 was not required for degradation of NCOA4 (Figure S4H), a previously established substrate of HERC2 that uses domains other than RLD3 for interaction.^31^ Consistent with this result, NCOA4 degradation was fully competent in HERC2 knockdown cells complemented with the ΔRLD3 mutant of HERC2, but not in cells complemented with the catalytically inactive HERC2(C4762S) (Figure S4I).

These results collectively demonstrate that HERC2-mediated degradation of endogenous CCT4 and overexpressed CCT4 reporter are strictly dependent on ZNRD2. Given the dependence on ZNRD2 for HERC2 interaction with CCT4, we conclude that ZNRD2 is an adaptor that recruits HERC2 to orphan CCT4. Other orphaned CCT subunits seemed likely to also use ZNRD2 as a HERC2 adaptor, given their interaction in affinity purification experiments (Figures 1E and S2). As was hinted by the effect of ZNRD2 knockdown on endogenous CCT7 (Figures 3B, S4A, and S4B), this extrapolation proved to be valid, as shown in further experiments described below.

### Reconstitution of orphaned CCT4 recognition and ubiquitination

**Figure S5:**
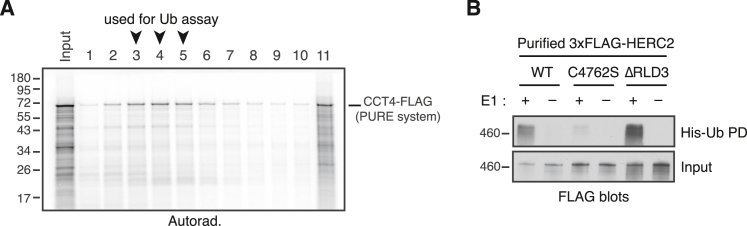
Additional characterization of *in vitro* ubiquitination assays, related to Figure 4 (A) ^35^S-methionine-labeled CCT4-3xFLAG was translated in a completely purified translation system (the PURE system; see method details) consisting of *E. coli* translation factors. The translation reaction was separated on a 5%–25% sucrose gradient and analyzed by SDS-PAGE and autoradiography. Soluble CCT4-3xFLAG migrating in fractions 3–5 (arrowheads) was recovered and used as the substrate for *in vitro* ubiquitination assays shown in Figure 4. Under the centrifugation conditions used, these fractions of the gradient would correspond to monomeric CCT4. A population of CCT4 fails to fold and is aggregated (fraction 11). (B) Purified WT 3xFLAG-HERC2 and its mutants (C4762S and ΔRLD3) were tested for auto-ubiquitination by incubation with E1, E2 (UBCH5), His-Ub, and ATP, as indicated. The reactions were analyzed by anti-FLAG immunoblotting, either directly (input; bottom) or after His-Ub pull-down under denaturing conditions (His-Ub PD; top). Auto-ubiquitination was observed for HERC2 WT and ΔRLD3, but not for catalytically inactive C4762S mutant. As expected, ubiquitination was dependent on E1 enzyme.

**Figure 4:**
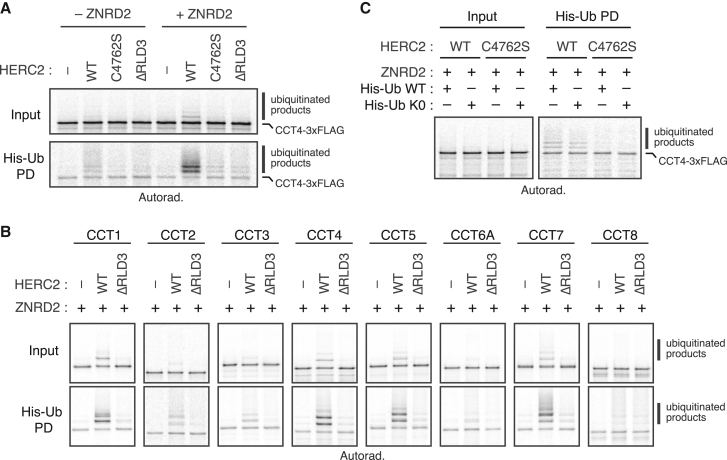
Reconstitution of CCT subunit ubiquitination with purified factors (A) ^35^S-methionine-labeled CCT4-3xFLAG was translated in the PURE system and the soluble and monomeric population was isolated (see Figure S5A). This was incubated with E1 and E2 enzymes, His-Ub, ATP, the indicated recombinant HERC2 variant, and ZNRD2, as indicated. The samples were analyzed directly (input) or after denaturing His-Ub pull-down (His-Ub PD). (B) Each CCT subunit was subjected to *in vitro* ubiquitination as in (A). (C) *In vitro* ubiquitination as in (A), with either His-Ub (WT) or its lysine-free mutant (K0). See also Figure S5.

The functional experiments in cells establish the requirement for HERC2 in the QC of multiple CCT subunits, with ZNRD2 serving as a necessary adaptor in the case of CCT4. To determine whether these components are sufficient to initiate the degradation pathway, we reconstituted substrate ubiquitination *in vitro*. Here, radioactively labeled CCT4 was produced by *in vitro* translation (IVT) using purified *E. coli*-derived translation factors and ribosomes (the so-called protein synthesis using recombinant elements [PURE] translation system^32^). Size fractionation by sucrose gradient sedimentation showed that CCT4 was soluble and monomeric (Figure S5A), consistent with its robust folding into a stable protein in this system. Incubation of the peak fractions from this gradient with purified ZNRD2, HERC2, the E2 enzyme UBCH5, E1 enzyme, ubiquitin, and ATP led to CCT4 ubiquitination (Figure 4A, lane 6).

Ubiquitination was strongly reduced by the omission of ZNRD2 from the reaction (lane 2) or by the use of the ΔRLD3 mutant (lanes 4 and 8), which retained catalytic activity as determined by auto-ubiquitination (Figure S5B). A similarly low ubiquitination was seen for the catalytically inactive HERC2(C4762S) mutant (lanes 3 and 7), with little or no ubiquitination observed when HERC2 was omitted (lanes 1 and 5). The residual ubiquitination seen in samples lacking ZNRD2 (e.g., lanes 2 and 4), or with HERC2 mutants (lanes 7 and 8), might be due to a combination of E3-independent ubiquitination by the E2 enzyme, promiscuous interactions made by the very large multi-domain HERC2, or contaminating E3 ligase(s) in the ZNRD2 or HERC2 preparations, both of which derive from mammalian cells. Nonetheless, the strong dependence on ZNRD2, RLD3, and C4762, each of which is also crucial for CCT4 degradation in cells, supports the conclusion that CCT4 can be specifically ubiquitinated by the HERC2-ZNRD2 complex in a physiologically relevant reaction.

Analysis of all eight CCT subunits in this *in vitro* system showed that most of them are targets for RLD3-dependent HERC2 ubiquitination, albeit to varying levels of efficiency (Figure 4B). The best substrates in this purified system were CCT1, 4, 5, and 7, with CCT2 and 3 showing lower but detectable levels of RLD3-dependent ubiquitination. CCT6A and CCT8, although detectably ubiquitinated at low levels, showed little or no RLD3-dependence. This is consistent with their minimal HERC2-dependent degradation in cells (Figure 2C) and lowest recovery of ZNRD2 in affinity purification experiments (Figures 1E and S2).

We noted that the primary ubiquitinated products for each of the ubiquitinated CCT subunits contained only one or two ubiquitins, and this was not impacted by the use of lysine-free ubiquitin in the reaction (Figure 4C). It therefore seems that the minimal ZNRD2-HERC2 system is sufficient for multi-monoubiquitination but not ubiquitin chain formation. An emerging theme in protein QC (PQC) is the use of different factors for the initiation and elongation steps leading to a polyubiquitinated substrate.^33^^,^^34^ Based on its direct physical interaction with the substrate and mono-ubiquitination in a reconstituted system, we conclude the ZNRD2-HERC2 complex triggers initiation of QC for several orphan CCTs. The later steps that culminate in orphan CCT degradation remain to be studied.

### Mechanism of orphan CCT recognition by the ZNRD2-HERC2 complex

**Figure 5:**
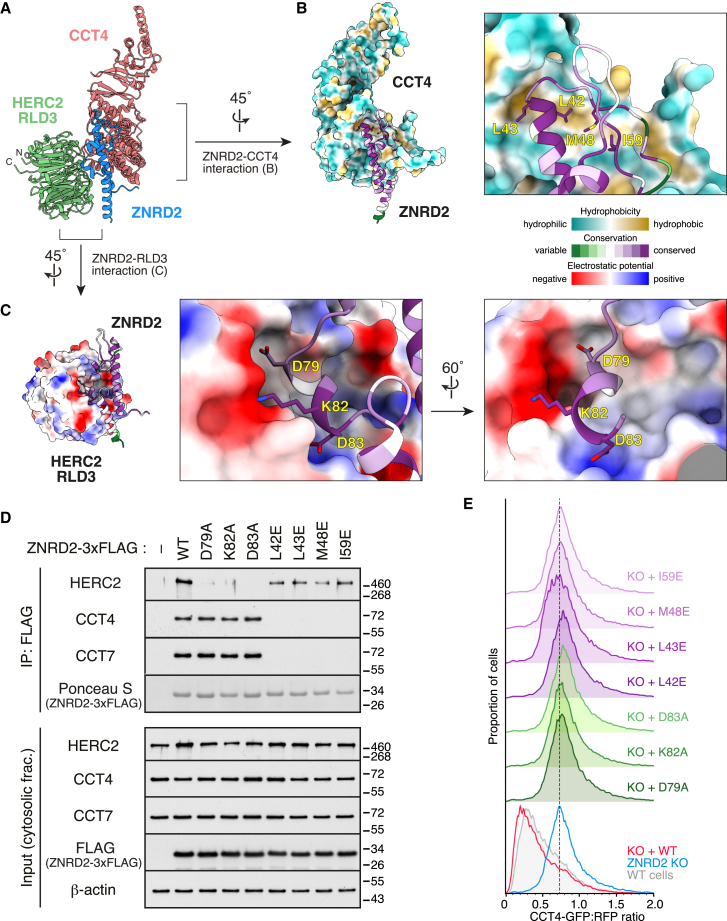
Structural basis of orphan recognition by the ZNRD2-HERC2 complex (A) AlphaFold-predicted model of a putative ternary complex consisting of CCT4 (pink), ZNRD2 (blue), and the RLD3 domain of HERC2 (green). The position of a C-terminal two-helix domain of ZNRD2 was not predicted with high confidence, so this element, the flexible linker attaching it to the N-terminal domain, and the unstructured N-terminal tail, are not shown. (B) Interface between CCT4 and ZNRD2 in the predicted model. CCT4 is shown in surface representation and colored by hydrophobicity. ZNRD2 is in cartoon representation and colored by conservation. The close-up view shows four highly conserved residues of ZNRD2 that occupy a hydrophobic patch of CCT4 also found in other CCT subunits. (C) Interface between ZNRD2 and RLD3 of HERC2 in the predicted model. RLD3 is shown in surface representation and colored by electrostatic potential, while ZNRD2 is shown as in (B). The close-up views show three highly conserved residues of ZNRD2 that mediate this predicted interaction. (D) ZNRD2-3xFLAG constructs containing point mutations in the residues, highlighted in (B) and (C), were transiently expressed in ZNRD2-KO cells and analyzed for interactions with endogenous HERC2, CCT4, and CCT7 by non-denaturing IP with anti-FLAG affinity resin. Input and IP samples were subjected to immunoblotting as indicated. (E) CCT4 reporter degradation was analyzed by flow cytometry in WT cells (filled gray), ZNRD2-KO cells (blue), and ZNRD2-KO cells transiently re-expressing WT (red) or mutant ZNRD2-3xFLAG constructs. The mutants are the same as those analyzed in (D). See also Figures S6 and S7.

**Figure S6:**
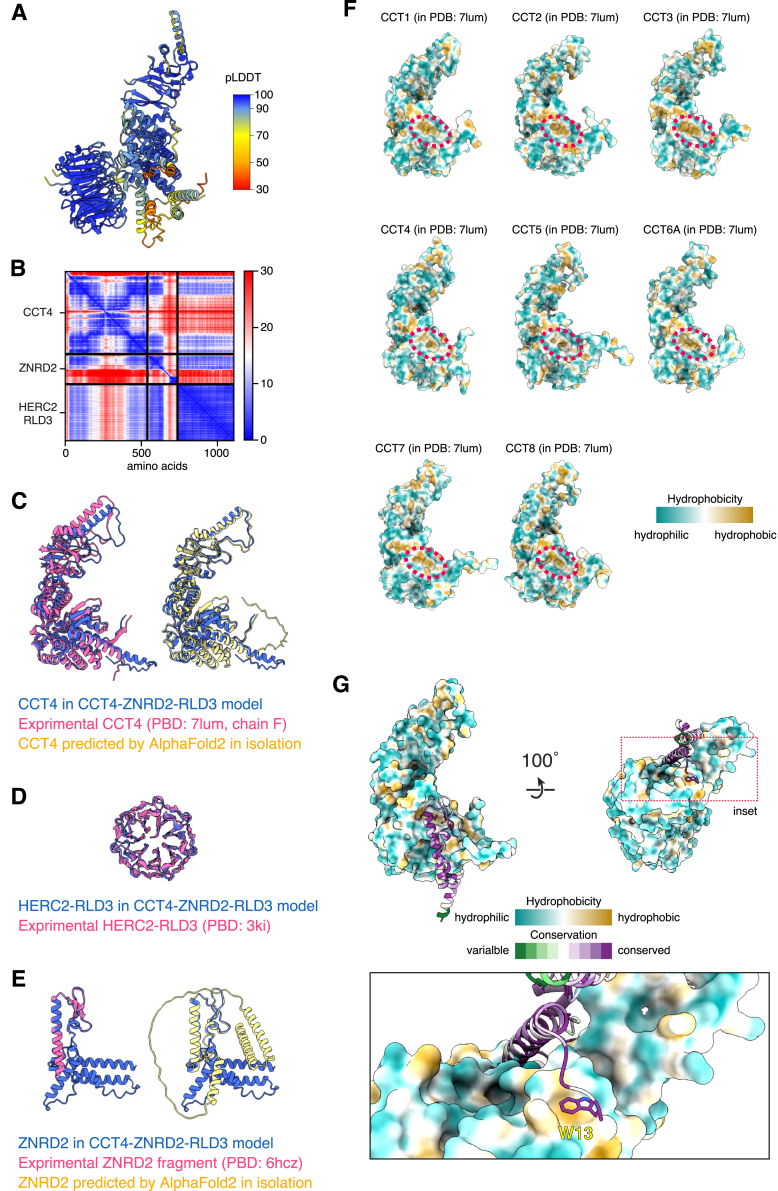
AlphaFold-predicted model of a putative CCT4-ZNRD2-HERC2 complex, related to Figure 5 (A and B) AlphaFold-predicted model (A) and predicted alignment error (PAE) plot (B) of a putative ternary complex consisting of CCT4, ZNRD2, and HERC2-RLD3. The predicted structural model is colored by per-residue confidence score (predicted local distance difference test [pLDDT]). (C) Comparison of CCT4 in the predicted CCT4-ZNRD2-RLD3 complex (blue) with its experimentally determined structure (pink; PDB: 7lum [chain F]) as well as with the model of CCT4 predicted by AlphaFold2 in isolation (yellow). (D) Comparison of RLD3 of HERC2 in the predicted CCT4-ZNRD2-RLD3 complex (blue) with its experimentally determined structure (pink; PDB: 3kci). (E) Comparison of ZNRD2 in the predicted CCT4-ZNRD2-RLD3 complex (blue) with experimentally determined structure of an N-terminal region of ZNRD2 (pink; PDB: 6hcz) as well as with the model of ZNRD2 predicted by AlphaFold2 in isolation (yellow). (F) Surface representation of individual CCT subunits obtained from an experimentally determined structure of the CCT complex (PDB: 7lum), colored by hydrophobicity. Red dashed circles indicate a hydrophobic patch shared by all CCT subunits. When engaged with ZNRD2, this hydrophobic patch is predicted to be occupied by four highly conserved residues of ZNRD2 (see Figure 5B). (G) Predicted interaction between the W13 residue of ZNRD2 and CCT4. CCT4 is shown as surface colored by hydrophobicity, while ZNRD2 is as cartoon colored by conservation. A close-up view highlighting the W13 residue of ZNRD2 that lies in a shallow hydrophobic patch of CCT4 is shown in the inset.

Having defined ZNRD2 and RLD3 of HERC2 as the minimal recognition module for orphaned CCT4, we used AlphaFold-Multimer^35^ to predict a putative ternary complex. High confidence interactions were predicted between ZNRD2 and both CCT4 and RLD3, but not between RLD3 and CCT4 (Figures 5A, S6A, and S6B). This matches the biochemical interaction data showing that ZNRD2 is an essential adaptor, motivating us to investigate this prediction further. In this structural model (which was validated by mutagenesis experiments described below), CCT4 and RLD3 are nearly identical to their individual predictions (Figures S6C and S6D) and consistent with expectations based on experimentally determined structures.^36^^,^^37^ By contrast, ZNRD2 is better ordered and more compact in the predicted complex than when its structure is predicted in isolation (Figure S6E).

In the model, ZNRD2 binds primarily to the surface of CCT4, which in fully assembled CCT would interact with CCT5 and CCT2 from the opposite ring. The majority of the interaction is mediated by a well-conserved helix in ZNRD2 (from E18 to R44) and its flanking domains (Figure 5B). Notably, the structural prediction of the CCT4-interacting region is nearly identical to the crystal structure of this domain^38^ (Figure S6E). The C-terminal region of this helix and flanking loop abut a conserved hydrophobic patch shared by all CCT subunits (Figures 5B and S6F). Four highly conserved residues of ZNRD2 (L42, L43, M48, and I59) occupy this conserved hydrophobic patch, contributed in CCT4 by I77, M81, V83, and A88. Flanking the other side of the CCT-interacting helix of ZNRD2 is a highly conserved tryptophan (W13) that lies flat in a shallow hydrophobic patch formed by I128 and M446 in CCT4 (Figure S6G). These interactions would be compatible with each CCT subunit, consistent with the mass spectrometry and co-IP analysis of CCT subunit orphans (Figures 1D, 1E, and S2).

On the other side of ZNRD2 from its CCT-interacting helix, a short helix from D79 to D83 engages the 7-bladed β-propeller of RLD3 from HERC2 (Figure 5C). The residues of ZNRD2 that mediate this predicted interaction (D79, K82, and D83) are highly conserved. Notably, the N and C termini of RLD3 reside on the opposite side of the surface that binds ZNRD2. This organization means that the flanking regions of HERC2, whose structure remains to be determined, would not interfere with the RLD3-ZNRD2 interaction.

ZNRD2 point mutants in any of the four key hydrophobic residues that are predicted to engage the hydrophobic patch on CCT4 completely abolished interactions with CCT4 without disrupting the ZNRD2-HERC2 interaction (Figure 5D). Conversely, each of three ZNRD2 point mutants at sites of predicted interaction with RLD3 sharply impaired HERC2 interaction without impairing interactions with CCT4 (Figure 5D). Importantly, none of these mutants affected ZNRD2 expression. Reconstitution of ZNRD2 knockout cells with each of these mutants showed that all of them were ineffective in restoring degradation of the orphan CCT4 reporter (Figure 5E).

**Figure S7:**
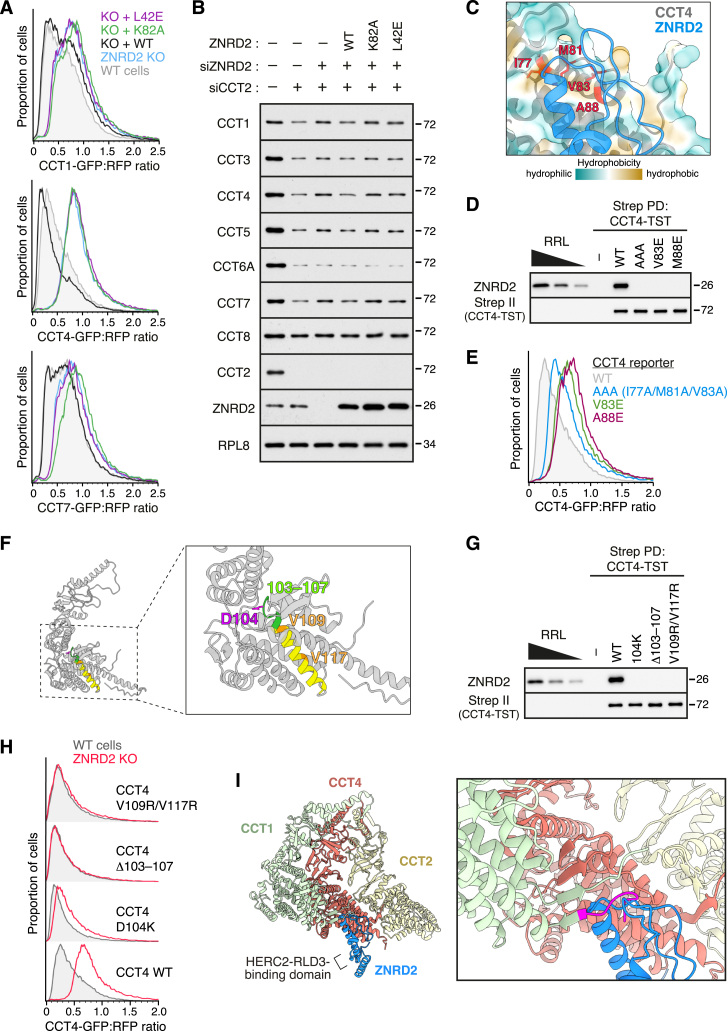
Recognition of orphan CCT subunits by ZNRD2, related to Figures 5 and 6 (A) The CCT1 (top), CCT4 (middle), and CCT7 (bottom) reporters were assessed for degradation by flow cytometry in WT cells, ZNRD2-KO cells (transfected with empty vector), and ZNRD2-KO cells transiently re-expressing WT or mutant ZNRD2 constructs, as indicated. (B) Cells were transfected with CCT2-targeting siRNA together with or without ZNRD2-targeting siRNA, as indicated. The following day, cells were transfected with GFP together with or without the indicated ZNRD2 constructs and then cultured for another 2 days. After sorting GFP-positive cells to enrich the transfected cells, total cell lysates were prepared and analyzed by immunoblotting for the indicated proteins. (C) Interface between CCT4 and ZNRD2 in the predicted CCT4-ZNRD2-RLD3 model. CCT4 is shown as black cartoon with transparent surface representation colored by hydrophobicity. ZNRD2 is shown in blue cartoon representation. Highlighted are the residues on the hydrophobic patch in CCT4 that forms part of the predicted ZNRD2 interaction surface. (D) The indicated CCT4-TST constructs were translated in RRL, affinity-purified under native conditions, and analyzed by immunoblotting as indicated. Three amounts of total RRL were analyzed in parallel. The AAA mutant contains I77A, M81A, and V83A mutations. (E) The indicated CCT4 reporter constructs were expressed in WT cells and assessed for degradation by flow cytometry. (F) Cartoon representation of CCT4 in the predicted CCT4-ZNRD2-RLD3 model. The inset shows a close-up view highlighting the residues deleted or mutated in the misfolding CCT4 mutants used in (G) and (H). (G) The indicated CCT4-TST constructs were translated in RRL, affinity-purified under native conditions, and analyzed by immunoblotting as indicated. Three amounts of total RRL were analyzed in parallel. (H) The indicated CCT4 reporter constructs were assessed for degradation by flow cytometry in WT and ZNRD2-KO cells. (I) AlphaFold-predicted model of the CCT4-ZNRD2-RLD3 complex was docked onto an experimentally determined structure of the CCT complex (PDB: 7lum) by aligning CCT4 in the prediction with one of the two CCT4 chains in the experimental structure. CCT1 (light green), CCT2 (light yellow), and CCT4 (light coral) from the experimentally determined structure, and ZNRD2 (blue) from the predicted model, are shown in cartoon representation. A close-up view shows that the N-terminal tail of CCT1 (magenta) might clash with ZNRD2 if it were to remain in the position seen in fully assembled CCT. Given the minimal interactions made by this tail (and the adjacent C-terminal tail), we posit that they could easily move out of the way to permit ZNRD2 binding to CCT4, even when CCT4 is flanked by adjacent CCT subunits.

We further showed that key mutants in ZNRD2 at the interface with CCT4 (L42E) and HERC2 (K82A) also impaired degradation of the CCT1 and CCT7 reporters (Figure S7A). Using these mutants, we tested the structural model on degradation of endogenous orphan CCTs. As seen with HERC2 knockdown (Figure 2C), acute knockdown of ZNRD2 partially impaired degradation of several CCT subunits orphaned by CCT2 knockdown (Figure S7B). As with HERC2, degradation of CCT6A and CCT8 was not affected by the presence or absence of ZNRD2, whereas CCT1, 4, 5, and 7 were the most responsive. Reconstitution of ZNRD2 knockdown cells with WT ZNRD2 restored CCT subunit degradation, in contrast to either the L42E or K82A mutants. Thus, all CCT orphans that use the ZNRD2-HERC2 pathway are recognized by a similar mechanism.

Based on the structural model, ZNRD2 recognition of orphaned CCT subunits relies on a hydrophobic patch on the CCT subunit that is generated upon its folding (Figures 5B, S6F, and S7C). Consistent with this idea, mutations that reduce the hydrophobicity of this patch on CCT4 led to loss of the ZRND2-CCT4 interaction *in vitro* (Figure S7D) and impaired degradation of the CCT4 reporter in cells (Figure S7E). Mutations in the buried core of folded CCT4 that would disrupt its folding also prevented interaction with ZNRD2 (Figures S7F and S7G). Unlike the structure-guided mutants to the hydrophobic patch, the misfolding mutants are efficiently degraded in both WT and ZNRD2 KO cells (Figure S7H). Thus, misfolded CCT4 is degraded by a (yet unidentified) misfolded PQC pathway, whereas folded but orphaned CCT4 is degraded by the ZNRD2-HERC2 pathway.

### Recognition of partial CCT assemblies by the ZNRD2-HERC2 complex

**Figure 6:**
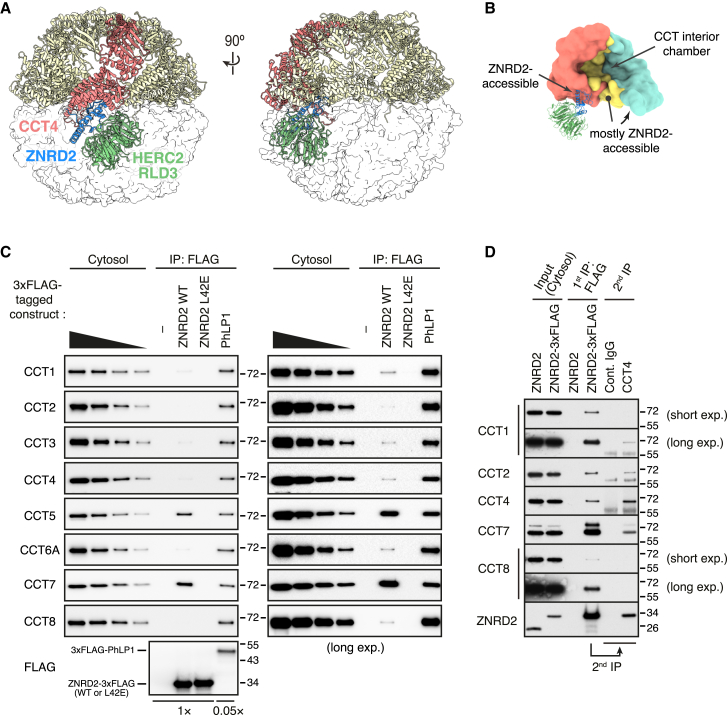
Recognition of partially assembled CCT subunits by the ZNRD2-HERC2 complex (A) The CCT4-ZNRD2-RLD3 model was docked into the complete CCT complex structure (PDB: 7lum) by aligning the CCT4 chains. The CCT4-ZNRD2-RLD3 model is colored, as in Figure 5A. The non-CCT4 subunits within the top ring of the CCT complex are shown as cartoons in khaki. The CCT subunits of the bottom ring are shown in transparent surface representation. (B) Cartoon depicting a hypothetical partial CCT assembly comprising three subunits (colored in light coral, yellow, and cyan). One of the end subunits is compatible with binding the ZNRD2-HERC2 complex. The interior chamber is indicated for reference. (C) HEK293T cells transiently overexpressing the indicated 3xFLAG-tagged proteins were subjected to non-denaturing anti-FLAG IP. The bound proteins were eluted with 3xFLAG peptide and subsequently analyzed by immunoblotting for the indicated proteins relative to serial 2-fold dilutions of the cytosolic fraction (cytosol) prepared from control transfected cells. 20-fold less of the 3xFLAG-PhLP1 sample was loaded on the gel relative to the control and ZNRD2-3xFLAG (WT and L42E) samples. Two exposures of the blots are shown for all CCT subunits. (D) ZNRD2-KO cells stably overexpressing ZNRD2-3xFLAG (or untagged ZNRD2 as a negative control) were subjected to non-denaturing anti-FLAG IP. The bound proteins were eluted with 3xFLAG peptide and subjected to a second round of non-denaturing IP with anti-CCT4 antibody or control IgG. Samples were analyzed by immunoblotting as indicated. See also Figure S7.

In the fully assembled CCT double ring, all potential ZNRD2 interaction sites are completely buried because they form the interface between the two individual rings (Figure 6A). This explains why fully assembled CCT would not be targeted by the ZNRD2-HERC2 QC system. By contrast, a partially assembled ring complex would contain a fully exposed ZNRD2 binding site on one of the terminal subunits (Figure 6B). Thus, the structural model predicts that ZNRD2 should be able to engage partial CCT assemblies.

We tested this idea using sequential co-IP. We first showed that affinity purification of overexpressed FLAG-tagged ZNRD2 co-purified each of the eight CCT subunits, albeit at very different relative amounts than found in the starting lysate (Figure 6C). By contrast, the CCT co-chaperone PhLP1, which engages fully assembled CCT,^39^ recovered all eight CCT subunits in the same stoichiometry as the starting lysate. No CCT subunits were recovered with ZNRD2(L42E), a point mutant at the predicted interface between ZNRD2 and CCT subunits. Thus, ZNRD2 does not engage CCT as a substrate, which would recover equal amounts of all CCT subunits and would not be prevented by the L42E mutation; rather, it engages unassembled CCT subunits.

To test whether some of these unassembled CCT subunits are in partially assembled complexes, we re-immunoprecipitated the natively eluted ZNRD2-associated products with anti-CCT4 (Figure 6D). This revealed that at least some proportion of CCT4 recovered by ZNRD2 is also associated with CCT1, CCT2, and CCT7 (probably as part of different complexes). The absence of CCT8 in this second IP further illustrates that we are not simply visualizing fully assembled CCT in these co-IPs. Thus, ZNRD2 can engage partially assembled CCT complexes and not just individual orphan CCT subunits.

The implication of this result is that failures at any of several steps along the (still poorly understood) CCT assembly pathway can be targeted for QC by the ZNRD2-HERC2 system. Although such partial intermediates seem to either be of low abundance or biochemically unstable (e.g., Figures 2D and 2F), they can evidently still access the ZNRD2-HERC2 QC system. Whether HERC2 can ubiquitinate subunits distal to the ZNRD2-interacting subunit is unknown, but even if it cannot, they would become accessible once the ubiquitinated subunit is extracted for degradation. Formation of a complete ring of CCT subunits may disfavor ZNRD2 binding because the N- and C-terminal tails of each CCT subunit would slot into the positions observed in the fully assembled structure. With these tails in place, the hydrophobic patch in each CCT subunit important for ZNRD2 binding is partially occluded (Figure S7I). The completed ring can then associate with a second ring to form the final CCT assembly. In this way, the ZNRD2-HERC2 system can engage any of several orphans and partial assemblies if they persist too long in the cytosol.

### Failure to degrade orphan CCT subunits impairs cell fitness

Genome-wide knockouts in over a thousand different cancer cell lines indicate that loss of either ZNRD2 or HERC2 impairs fitness in most of them, in some cases severely (Cancer Dependency Map; https://depmap.org/).^40^ To test more precisely the physiologic relevance of the QC pathway for orphan CCTs, we took advantage of the structure-guided ZNRD2 mutants that selectively disrupt its interaction with either CCT subunits or HERC2. We compared the relative fitness of ZNRD2 knockout cells, complemented with WT vs. mutant variants integrated into the same locus.

**Figure 7:**
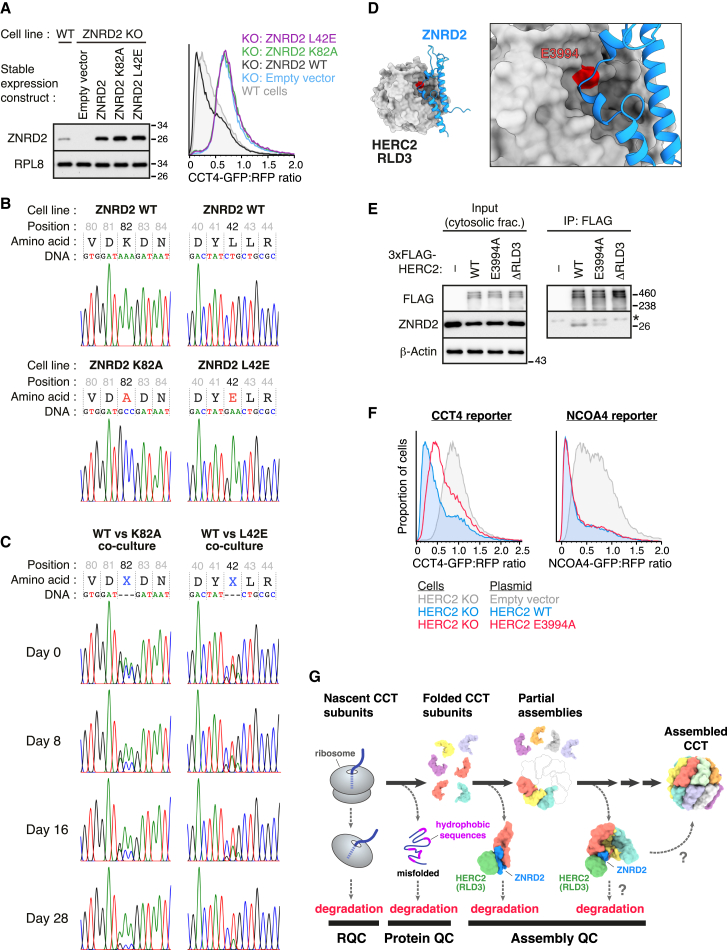
Physiological relevance of the HERC2-ZNRD2 pathway (A) ZNRD2-KO Flp-In T-REx 293 cells were complemented with either empty vector or the indicated ZNRD2 constructs at the unique FRT site in their genome. These cells were transfected with the CCT4 reporter and analyzed by immunoblotting (left) and flow cytometry (right). Wild-type (WT) Flp-In T-REx 293 cells were analyzed in parallel for comparison. (B) The exogenous ZNRD2 sequence at the FRT site was PCR-amplified from the genomic DNA of ZNRD2-KO cells complemented with ZNRD2 WT or its mutants (K82A or L42E), and the amplicons were sequenced. Shown are sequencing chromatograms for the WT and mutant cells at the regions around the K82 and K42 codons. (C) ZNRD2-KO cells expressing ZRND2 WT were mixed at a ratio of 1:1 with ZNRD2-KO cells expressing either the K82A mutant (left; WT vs. K82A) or the L42E mutant (right; WT vs. L42E) and then cultured for 4 weeks. An aliquot of each co-culture was sampled at the indicated time points, and the exogenous ZNRD2 sequences at the FRT site were amplified and sequenced as in (B). Sequencing chromatograms around the K82 and L42 codons are shown for the co-cultures containing the respective mutant cell lines. (D) Interface between ZNRD2 and RLD3 of HERC2 in the predicted structural model. RLD3 is shown in gray surface representation, while ZNRD2 is in blue cartoon representation. The close-up view highlights the E3994 residue (red) of HERC2. (E) HEK293T cells transiently expressing the indicated 3xFLAG-HERC2 constructs were subjected to anti-FLAG IP under non-denaturing conditions. Input and IP samples were analyzed by immunoblotting for the indicated proteins. The asterisk indicates a cross-reactive nonspecific band. (F) HERC2-KO cells were transfected with the CCT4 reporter (left) or the NCOA4 reporter (right), together with the indicated plasmids, and analyzed by flow cytometry. (G) Model for quality control (QC) during CCT biogenesis. Depending on the step at which biogenesis fails, qualitatively different types of QC act on the substrate. This includes ribosome-associated quality control (RQC) if CCT translation fails, protein QC if a CCT subunit fails to fold after translation, and assembly QC for several CCT subunits that fold but do not assemble into the CCT. Unassembled CCT subunits can be recognized by ZNRD2, which engages the RLD3 domain of the E3 ligase HERC2, and ubiquitinates the subunit(s) to target it for degradation. ZNRD2 can also recognize partially assembled complexes, but the triage of these complexes between degradation and assembly remains to be investigated. ZNRD2 cannot engage assembled CCT because its interaction surfaces are buried.

These stable cell lines modestly overexpress the exogenous ZNRD2 variants to equal levels, with the mutants being completely inactive for CCT4 reporter degradation (Figure 7A). Each mutant-overexpressing cell line was mixed in equal amounts with the matched WT overexpressing cell line, then co-cultured for 4 weeks. Whereas the alleles were equally represented at the start of the co-culture, the WT allele progressively dominated over each mutant (Figures 7B and 7C). Thus, disrupting either the ZNRD2-CCT subunit interaction or the ZNRD2-HERC2 interaction compromises fitness, strongly suggesting that the orphan CCT degradation pathway is crucial for optimal cell homeostasis.

At the organism level, HERC2 is essential in mouse, with the heterozygote showing a neurodegeneration phenotype. However, the aspects of this phenotype that are contributed by a failure of orphan CCT subunit degradation is unclear. With knowledge of the specific region of HERC2 involved in ZNRD2 binding, we searched the human disease-linked database (ClinVar; https://www.ncbi.nlm.nih.gov/clinvar/) for a more precise perturbation. The E3994A mutant, found in a patient with neurodevelopmental disorders, was located in RLD3 close to the site of ZNRD2 interaction (Figure 7D). This mutant showed reduced interaction with ZNRD2 by co-IP and reduced capacity to trigger CCT4 reporter degradation in cells (Figures 7E and 7F). Importantly, the mutation had no effect on expression levels of HERC2 and no impairment in its ability to trigger degradation of NCOA4. Thus, HERC2(E3994A) is a functional E3 ligase that is selectively impaired in the ZNRD2 interaction critical for degradation of orphan CCTs. Disease causality by E3994A should be interpreted with some caution, given the single patient. Nonetheless, the clear cellular phenotype seen with this mutant, the fitness cost in cells seen with selective perturbation of the ZNRD2-HERC2 interaction, and a similar disease phenotype as a well-established hypomorphic HERC2 allele all argue for a causal link with human disease.

## Discussion

We have identified ZNRD2 and HERC2, two little-studied proteins, as key factors required for efficient recognition and elimination of several unassembled CCT subunits. QC of protein complex assembly is not well understood,^4^^,^^5^ so discovery of the factors that monitor one of the cell’s essential and most abundant complexes provides a paradigm that will guide future work in this area. We then leveraged this discovery and recent advances in structure prediction to define and validate the molecular basis of orphan CCT recognition. Although numerous QC factors have been described,^41^ the precise mechanism of recognition is generally ill-defined.^42^ The CCT4-ZNRD2-HERC2 interaction represents an unprecedented molecular snapshot of how incompletely assembled complexes are recognized. The concept that emerges is that QC can operate on correctly folded proteins, a departure from the long-standing paradigm of QC via misfolded linear polypeptide segments.

### Three modes of target recognition during QC

QC is used at multiple stages of protein biosynthesis, each of which uses a qualitatively different mechanism of substrate recognition (Figure 7G). The most widely studied, generally termed PQC, involves post-translational recognition of proteins that fail to fold or localize correctly.^41^ Substrate recognition during PQC typically operates on linear segments of polypeptide containing hydrophobic sequences^43^^,^^44^ or, in the case of membrane proteins, hydrophilic segments exposed within the membrane.^45^ These regions are normally buried or removed in the mature protein. During ribosome-associated QC (RQC), which acts during translation, the nascent protein is not involved in recognition. Instead, the QC factors recognize particular configurations of ribosomes, such as an empty A site or the interface between two collided ribosomes.^46^

Our findings suggest that assembly QC (AQC) of CCT targets correctly folded substrates, distinguishing this mechanism of recognition from either PQC or RQC. Evidence for this idea comes from four findings. First, a substantial proportion of each CCT subunit produced in the PURE translation system is soluble and monomeric, and this population can be targeted by the ZNRD2-HERC2 system for six of eight subunits. Proteins that fail to fold in this chaperone-free translation system readily aggregate,^14^^,^^28^ so CCT subunit solubility suggests it is folded. Second, the mutagenesis-validated structural prediction shows that the ZNRD2 interaction surface is on folded CCT4. Furthermore, the domain of ZNRD2 that engages CCT4 does not contain any grooves or pockets that are typically used to bind linear polypeptide. Third, mutagenesis of the hydrophobic surface on folded CCT4 that is predicted to engage ZNRD2 abolishes its degradation, whereas misfolded CCT4 is degraded by a ZNRD2-independent pathway. Fourth, ZRND2 can target assembly competent CCT subunits, as judged by its interaction with partially assembled CCT complexes. This implies that ZNRD2 recognizes CCT subunits capable of forming lateral interactions with partners, which likely requires folding of the interacting subunits. The combined evidence argues strongly that folded CCT orphans are targets of AQC.

### Recognition of folded proteins for degradation

Two observations suggest that QC of folded proteins may be a general principle. First, the QC factor UBE2O recognizes unassembled α-hemoglobin subunits via an interface that is normally shielded by an assembly factor termed AHSP.^27^^,^^28^ Although the mechanism of UBE2O interaction with α-hemoglobin remains to be elucidated, the fact that AHSP binds to folded α-haemoglobin^47^ implies that UBE2O recognition is similar. Second, inappropriate heterodimers of BTB domains, although folded, can be recognized by FBXL17 to trigger disassembly and ubiquitination.^48^ Such assemblies are not orphans per se, but do reflect the principle that misfolding is not the sole, or perhaps even the major, target of QC.

Orphan recognition independent of misfolding might be especially important for proteins, such as ATPases, whose folding is robust even in the absence of complex formation. Thus, many protein complexes might use qualitatively different mechanisms of recognition than those used by misfolded proteins. Direct recognition of folded but unassembled proteins has the advantage of disposing them before they unfold in the absence of their partner(s). Relying on unfolding for orphan recognition imposes a risk of aggregation, so pre-empting this possibility would provide potential benefits to the cell, especially for highly abundant or particularly aggregation-prone proteins.

### QC by the HERC family of ligases

The discovery of HERC2 as overseeing assembly failures in CCT is directly analogous to the recent finding that HERC1 monitors failures during ATPase ring assembly of the 19S proteasome.^14^ Although the molecular details of target recognition by HERC1 remain to be elucidated, two important parallels are noteworthy. First, both HERC1 and HERC2 use adaptors for recognition of their ATPase substrates. Second, the domain that interacts with the adaptor in both cases is a β-propeller domain that is widely used for protein-protein interactions.

β-propellers are found in each of the six HERC family members.^49^ HERC1 and HERC2 both have three β-propeller domains, with each of the remaining HERCs having one. Based on the specificity of HERC1’s RLD1 for PAAF1 and HERC2’s RLD3 for ZNRD2, it seems likely that each of the other eight β-propellers engages a different target. This would imply that the HERCs as a family could serve as QC factors for numerous biological processes via recruitment of different types of adaptor proteins. Our findings suggest that proteomic analysis of interactors for each β-propeller could be used to systematically uncover adaptors (e.g., Figure 3F) that are then be used to find substrates (e.g., Figure 6C).

### Implications for protein complex assembly

In the case of HERC1, the adaptor for target recognition is an established 19S assembly factor termed PAAF1. It was proposed that prolonged retention of the assembly factor on partial ATPase ring assemblies signifies failure, favoring HERC1 recruitment.^14^ Extrapolation of this concept to HERC2 implies that ZNRD2 might be an assembly factor for CCT, an idea that warrants future investigation.

The observation that ZNRD2 can interact with most CCT subunits, can engage partial assemblies, and shields some but not other subunit-subunit interaction surfaces is consistent with a possible role in assembly. It is noteworthy that a key surface shielded by ZNRD2 is the inter-ring interface, indicating that a partially assembled ring with bound ZNRD2 cannot associate with another ring. This is directly analogous to PAAF1 and other chaperones for the 19S ATPase ring, binding of which precludes assembly with 20S rings. Thus, the mechanistic linking of AQC with productive assembly via assembly factors serving as adaptors for ubiquitination machinery might be a general principle for multi-protein complexes. This insight provides several routes to understanding both assembly and AQC.

### Physiologic relevance of AQC

AQC is likely to be most important for cell types that are exceptionally long-lived, such as mammalian neurons, and need to avoid age-related proteotoxicity.^50^ Indeed, a rather subtle hypomorphic allele of HERC1 that is deficient in PAAF1 recognition causes severe neurodegeneration in mice.^14^^,^^51^^,^^52^^,^^53^ Similarly, mice lacking one HERC2 allele show neuronal phenotypes with evidence of perturbed proteostasis and loss of cerebellar Purkinje cells.^54^ In humans, a hypomorphic allele of HERC2 that leads to reduced expression causes a neurodevelopmental disease^55^^,^^56^ that overlaps substantially with the phenotypes seen in a patient with HERC2(E3994A) at the interaction site with ZNRD2.

At the cellular level, a fitness cost was seen for cells carrying a point mutation in ZNRD2 that selectively impairs its interaction with either HERC2 or CCT subunits, showing that disruption of even one AQC pathway is detrimental. Aneuploid cancer cells may be particularly dependent on AQC pathways given their high burden of orphans.^57^^,^^58^^,^^59^^,^^60^ Protein subunits that are either highly abundant or highly toxic would have imposed particularly strong selective pressure to evolve and maintain dedicated AQC pathways.

AQC pathways are only now being uncovered because they are mostly absent from the simpler yeast system used for most prior work on QC. Their absence in yeast and other unicellular organisms could be because protein aggregates are more readily resolved to confer stress-tolerance^61^ and because the aggregates can be jettisoned to the mother cell during cell division.^62^^,^^63^ By comparison, the complex regulation of gene expression in metazoans, with their much larger genomes and need to differentially regulate proteins across various cell types, may incur greater noise and imbalances in subunit stoichiometry. This might explain why orphan subunits seem to be the largest source of degraded proteins in mammalian cells,^13^^,^^14^ thereby necessitating robust AQC. We anticipate this field to be a rich direction of study, with substantial implications for our understanding of cellular homeostasis and for diseases of proteotoxicity, aging, and cancer.

### Limitations of the study

The ZNRD2-HERC2 pathway is needed for elimination of many but not all unassembled CCT subunits. In particular, the alternative pathways used to eliminate orphaned CCT3 and CCT6 remain to be determined. The ZNRD2-HERC2 pathway adds monoubiquitin(s) to its CCT substrates. Whether this is sufficient for degradation or requires subsequent ubiquitin chain formation by another factor(s) is not known. We show that ZNRD2 is capable of recognizing partially assembled CCT complexes. The composition of these partial assemblies and their subsequent fates remain to be elucidated. It is possible that these partial assemblies are assembly intermediates, that they are dead-end products, or a combination of both. Whether HERC2 ubiquitinates all the subunits in these partial assemblies or only the subunit that is directly recognized by ZNRD2 has not been determined. Roles for ZNRD2 and HERC2 beyond AQC of CCT have not been explored. It is possible that ZNRD2 participates in CCT assembly by serving as a chaperone that temporarily shields sensitive surfaces on CCT subunits. HERC2 probably has other unidentified substrates that could be recognized using domains other than RLD3. The full complement of HERC2 substrates remains to be determined. The E3994A mutation in the RLD3 domain of HERC2 was found in a single patient, so its causal role in the patient’s neurodevelopmental disorders should be considered provisional.

## STAR★Methods

### Key resources table

**Table:** 

REAGENT or RESOURCE	SOURCE	IDENTIFIER
**Antibodies**		

Rabbit polyclonal anti-TCP1	Proteintech	Cat#10320-1-AP; RRID:AB_10694136
Rabbit polyclonal anti-TCP1	Bethyl	Cat#A303-445A; RRID:AB_10948976
Rabbit polyclonal anti-CCT2	Bethyl	Cat#A303-479A; RRID:AB_10950578
Rabbit polyclonal anti-CCT3	Bethyl	Cat#A303-458A; RRID:AB_10950575
Rabbit polyclonal anti-CCT4	Bethyl	Cat#A304-725A; RRID:AB_2620920
Rabbit polyclonal anti-CCT4	Bethyl	Cat#A304-726A; RRID:AB_2620921
Rabbit polyclonal anti-CCT6A	Novus	Cat#NBP2-46715
Rabbit polyclonal anti-CCT7	Bethyl	Cat#A304-730A; RRID:AB_2620925
Rabbit polyclonal anti-CCT8	Proteintech	Cat#12263-1-AP; RRID:AB_906331
Rabbit polyclonal anti-HERC1	Bethyl	Cat#A301-904A; RRID:AB_1524073
Rabbit polyclonal anti-HERC2	Bethyl	Cat#A301-905A; RRID:AB_1524099
Rabbit polyclonal anti-HECTD3	Bethyl	Cat#A304-924A; RRID:AB_2621118
Rabbit polyclonal anti-ZNRD2	Proteintech	Cat#12273-1-AP; RRID:AB_2195902
Rabbit polyclonal anti-Strep-tag II	Abcam	Cat#ab76949; RRID:AB_1524455
Rabbit monoclonal anti-Strep-tag II	Abcam	Cat#ab180957
Rabbit monoclonal anti-RPL8 (uL2)	Abcam	Cat#ab169538; RRID:AB_2714187
Mouse monoclonal anti-CCT5	Proteintech	Cat#67400-1-Ig; RRID:AB_2882643
Mouse monoclonal anti-CCT7	Proteintech	Cat#67540-1-Ig; RRID:AB_2882759
Mouse monoclonal anti-CCT8	Proteintech	Cat#67539-1-Ig; RRID:AB_2882758
Mouse monoclonal anti-ZNRD2	Novus	Cat#NBP2-03198
Mouse monoclonal anti-FLAG	Sigma-Aldrich	Cat#F3165; RRID:AB_259529
HRP-conjugated mouse monoclonal anti-FLAG	Sigma-Aldrich	Cat#A8592; RRID:AB_439702
HRP-conjugated mouse monoclonal anti-β-actin	Sigma-Aldrich	Cat#A3854; RRID:AB_262011
HRP-conjugated goat polyclonal anti-rabbit IgG (H + L)	Jackson ImmunoResearch Labs	Cat#111-035-003; RRID:AB_2313567
HRP-conjugated goat polyclonal anti-mouse IgG (H + L)	Jackson ImmunoResearch Labs	Cat#115-035-003; RRID:AB_10015289
HRP-conjugated mouse monoclonal anti-rabbit IgG, light chain-specific	Proteintech	Cat#SA00001-7L; RRID:AB_2890988
Clean-Blot IP Detection Reagent (HRP)	Thermo Fisher Scientific	Cat#21230; RRID:AB_2864363
Anti-FLAG M2 affinity gel	Sigma-Aldrich	Cat#A2220; RRID:AB_10063035

**Chemicals, peptides, and recombinant proteins**		

Blasticidin S	Santa Cruz Biotechnology	Cat#sc-204655; CAS: 3513-03-9
Hygromycin B	Sigma-Aldrich	Cat#400051; CAS: 31282-04-9
Zeocin	Thermo Fisher Scientific	Cat#R25001; CAS: 11006-33-0
PEI MAX - Transfection Grade	Polysciences	Cat#24765; CAS: 49553-93-7
Doxycycline	Sigma-Aldrich	Cat#D9891; CAS: 24390-14-5
Digitonin, High Purity	Millipore	Cat#300410; CAS: 11024-24-1
cOmplete, EDTA-free Protease Inhibitor Cocktail	Roche	Cat#11873580001
3xFLAG Peptide	Sigma-Aldrich	Cat#F4799
Recombinant human His-Ubiquitin	Boston Biochem	Cat#U-530
Recombinant human His-Ubiquitin Mutant No K (K0)	Boston Biochem	Cat#UM-HNOK
Recombinant human GST-UBE1	Boston Biochem	Cat#E-306
Recombinant human UBCH5a	Boston Biochem	Cat#E2-616
Recombinant human 3xFLAG-HERC2	This paper	N/A
Recombinant human 3xFLAG-HERC2(C4762S)	This paper	N/A
Recombinant human 3xFLAG- HERC2ΔRLD3	This paper	N/A
Recombinant human ZNRD2-3xFLAG	This paper	N/A
Recombinant RNasin Ribonuclease Inhibitor	Promega	Cat#N2518
SP6 RNA Polymerase	New England Biolabs	Cat#M0207
CAP (7-methyl diguanosine triphosphate cap structure analog)	New England Biolabs	Cat#S1404L
S7 Micrococcal Nuclease	Roche	Cat#10107921001
Creatine Kinase	Roche	Cat#10127566001
Creatine phosphate	Roche	Cat#10621714001; CAS:71519-72-7
Amino acid kit	Sigma-Aldrich	Cat#09416
EasyTag L-[^35^S]-Methionine	Perkin Elmer	Cat#NEG709A005MC
Ponceau S solution	Sigma-Aldrich	Cat#P-7170; CAS: 6226-79-5

**Critical commercial assays**		

DNeasy Blood & Tissue Kit	QIAGEN	Cat#69504
PURExpress In Vitro Protein Synthesis Kit	New England Biolabs	Cat#E6800S
PURExpress Δ (aa, tRNA) Kit	New England Biolabs	Cat#E6840S

**Deposited data**		

Proteomics data	This paper	PRIDE: PXD042773
Cryo-EM structure: human TRiC, closed state	Knowlton et al.^36^	PDB: 7LUM
Crystal structure: N-terminal region of human ZNRD2	Perdreau-Dahl et al.^38^	PDB: 6HCZ
Crystal structure: RLD3 of human HERC2	Walker et al.^37^	PDB: 3KCI
AlphaFold2-predicted model of human CCT4 (P50991)	Varadi et al.^64^	AF-P50991-F1-model_v2; https://alphafold.ebi.ac.uk
AlphaFold2-predicted model of human ZNRD2 (O60232)	Varadi et al.^64^	AF-O60232-F1-model_v2; https://alphafold.ebi.ac.uk

**Experimental models: Cell lines**		

Human: HEK293T	ATCC	ATCC CRL-3216; RRID:CVCL_0063
Human: Flp-In T-REx 293	Thermo Fisher Scientific	Cat#R78007; RRID:CVCL_U427
Human: Expi293F	Thermo Fisher Scientific	Cat#A14527; RRID:CVCL_D615
Human: MCF7	AstraZeneca Global Cell Bank	AstraZeneca Global Cell Bank ID: 76305
Flp-In T-REx 293: Tet-On:: Empty vector	This paper	Internal ID: cYY031
Flp-In T-REx 293: Tet-On:: SF-HERC2	This paper	Internal ID: cYY035
Flp-In T-REx 293: Tet-On:: SF-HERC2(C4762S)	This paper	Internal ID: cYY033
HERC2-KO Flp-In T-REx 293	This paper	Internal ID: cYY030-13
ZNRD2-KO Flp-In T-REx 293	This paper	Internal ID: cYY023-27
ZNRD2-KO Flp-In T-REx 293: Empty vector	This paper	Internal ID: cYY024
ZNRD2-KO Flp-In T-REx 293: ZNRD2	This paper	Internal ID: cYY025
ZNRD2-KO Flp-In T-REx 293: ZNRD2-3xFLAG	This paper	Internal ID: cYY026
ZNRD2-KO Flp-In T-REx 293: CMVd3:: ZNRD2	This paper	Internal ID: cYY040
ZNRD2-KO Flp-In T-REx 293: CMVd3:: ZNRD2(K83A)	This paper	Internal ID: cYY043
ZNRD2-KO Flp-In T-REx 293: CMVd3:: ZNRD2(L42E)	This paper	Internal ID: cYY046

**Oligonucleotides**		

Silencer Select Negative Control siRNA #1	Thermo Fisher Scientific	Cat#4390843
Silencer Select siRNA against human CCT2 #1	Thermo Fisher Scientific	Cat#4392420; siRNA ID: s20756
Silencer Select siRNA against human CCT2 #2	Thermo Fisher Scientific	Cat#4392420; siRNA ID: s20757
Silencer Select siRNA against human HERC2 #1	Thermo Fisher Scientific	Cat#4392420; siRNA ID: s17063
Silencer Select siRNA against human HERC2 #2	Thermo Fisher Scientific	Cat#4392420; siRNA ID: s17064
Silencer Select siRNA against human HERC2 #3	Thermo Fisher Scientific	Cat#4392420; siRNA ID: s17062
Silencer Select siRNA against human ZNRD2 #1	Thermo Fisher Scientific	Cat#4392420; siRNA ID: s20655
Silencer Select siRNA against human ZNRD2 #2	Thermo Fisher Scientific	Cat#4392420; siRNA ID: s225439
Silencer Select siRNA against human ZNRD2 #3	Thermo Fisher Scientific	Cat#4392420; siRNA ID: s20654
Silencer Select siRNA against human HERC1 #1	Thermo Fisher Scientific	Cat#4392420; siRNA ID: s17065
Silencer Select siRNA against human HERC1 #2	Thermo Fisher Scientific	Cat#4392420; siRNA ID: s17066
gRNA targeting exon 10 of human *HERC2* gene: 5’-AAGCTCGTTGTCTTGTGGAA-3’	This paper	N/A
gRNA targeting exon 2 of human *ZNRD2* gene: 5’-ATGGGCGACTATCTGCTGCG-3’	This paper	N/A
Primer for genomic PCR and sequencing; gPCR-Z-F, 5’-CTGCTTCGCGATGGGCGGTAG-3’	This paper	N/A
Primer for genomic PCR: gPCR-Z-R, 5’-TCTAGACTCG AGTTATCAATGCTGCAAC-3’	This paper	N/A

**Recombinant DNA**		

pSP64: CCT1-TwinStrep	This paper	Internal ID: pYY214
pSP64: CCT2-TwinStrep	This paper	Internal ID: pYY221
pSP64: CCT3-TwinStrep	This paper	Internal ID: pYY212
pSP64: CCT4-TwinStrep	This paper	Internal ID: pYY222
pSP64: CCT4(D104K)-TwinStrep	This paper	Internal ID: pYY397
pSP64: CCT4(Δ103107)-TwinStrep	This paper	Internal ID: pYY399
pSP64: CCT4(V109R/V117R)-TwinStrep	This paper	Internal ID: pYY400
pSP64: CCT4(I77A/M81A/V83A [also referred to as AAA])-TwinStrep	This paper	Internal ID: pYY403
pSP64: CCT4(V83E)-TwinStrep	This paper	Internal ID: pYY407
pSP64: CCT4(A88E)-TwinStrep	This paper	Internal ID: pYY408
pSP64: CCT5-TwinStrep	This paper	Internal ID: pYY223
pSP64: CCT6A-TwinStrep	This paper	Internal ID: pYY224
pSP64: CCT7-TwinStrep	This paper	Internal ID: pYY225
pSP64: CCT8-TwinStrep	This paper	Internal ID: pYY226
pSP64: CCT3-TEV-3xFLAG	This paper	Internal ID: pYY213
pSP64: CCT7-TEV-3xFLAG	This paper	Internal ID: pYY219
PURE: CCT1	This paper	Internal ID: pYY286
PURE: CCT2	This paper	Internal ID: pYY287
PURE: CCT3	This paper	Internal ID: pYY288
PURE: CCT4	This paper	Internal ID: pYY289
PURE: CCT5	This paper	Internal ID: pYY290
PURE: CCT6A	This paper	Internal ID: pYY291
PURE: CCT7	This paper	Internal ID: pYY292
PURE: CCT8	This paper	Internal ID: pYY293
PURE: CCT4-TEV-3xFLAG	This paper	Internal ID: pYY281
pcDNA5/FRT/TO Vector	Thermo Fisher Scientific	Cat#V652020
pOG44 Flp-Recombinase Expression Vector	Thermo Fisher Scientific	Cat#V600520
pcDNA5/FRT/TO: CCT1-TEV-3xFLAG	This paper	Internal ID: pYY239
pcDNA5/FRT/TO: CCT2-TEV-3xFLAG	This paper	Internal ID: pYY233
pcDNA5/FRT/TO: CCT3-TEV-3xFLAG	This paper	Internal ID: pYY232
pcDNA5/FRT/TO: CCT4-TEV-3xFLAG	This paper	Internal ID: pYY234
pcDNA5/FRT/TO: CCT5-TEV-3xFLAG	This paper	Internal ID: pYY235
pcDNA5/FRT/TO: CCT6A-TEV-3xFLAG	This paper	Internal ID: pYY236
pcDNA5/FRT/TO: CCT7-TEV-3xFLAG	This paper	Internal ID: pYY237
pcDNA5/FRT/TO: CCT8-TEV-3xFLAG	This paper	Internal ID: pYY238
pcDNA5/FRT/TO: GFP-P2A-RFP	Chitwood et al.^65^	N/A
pcDNA5/FRT/TO: CCT1-GFP-P2A-RFP	This paper	Internal ID: pYY247
pcDNA5/FRT/TO: CCT2-GFP-P2A-RFP	This paper	Internal ID: pYY241
pcDNA5/FRT/TO: CCT3-GFP-P2A-RFP	This paper	Internal ID: pYY211
pcDNA5/FRT/TO: CCT4-GFP-P2A-RFP	This paper	Internal ID: pYY242
pcDNA5/FRT/TO: CCT4(D104K)-GFP-P2A-RFP	This paper	Internal ID: pYY436
pcDNA5/FRT/TO: CCT4(Δ103107)-GFP-P2A-RFP	This paper	Internal ID: pYY437
pcDNA5/FRT/TO: CCT4(V109R/V117R)-GFP-P2A-RFP	This paper	Internal ID: pYY438
pcDNA5/FRT/TO: CCT4(I77A/M81A/V83A [also referred to as AAA])-GFP-P2A-RFP	This paper	Internal ID: pYY439
pcDNA5/FRT/TO: CCT4(V83E)-GFP-P2A-RFP	This paper	Internal ID: pYY442
pcDNA5/FRT/TO: CCT4(A88E)-GFP-P2A-RFP	This paper	Internal ID: pYY443
pcDNA5/FRT/TO: CCT5-GFP-P2A-RFP	This paper	Internal ID: pYY243
pcDNA5/FRT/TO: CCT6A-GFP-P2A-RFP	This paper	Internal ID: pYY244
pcDNA5/FRT/TO: CCT7-GFP-P2A-RFP	This paper	Internal ID: pYY245
pcDNA5/FRT/TO: CCT8-GFP-P2A-RFP	This paper	Internal ID: pYY246
pcDNA5/FRT/TO: NCOA4-GFP-P2A-RFP	This paper	Internal ID: pYY274
pcDNA SF-HERC2 F1 (aa 1–969)	Chan et al.^66^	Addgene #55784; RRID:Addgene_55784
pcDNA SF-HERC2 F2 (aa 950–1750)	Chan et al.^66^	Addgene #55785; RRID:Addgene_55785
pcDNA SF-HERC2 F3 (aa 1700–2700)	Chan et al.^66^	Addgene #55786; RRID:Addgene_55786
pcDNA SF-HERC2 F4 (aa 2600–3600)	Chan et al.^66^	Addgene #55787; RRID:Addgene_55787
pcDNA SF-HERC2 F5 (aa 3550–4450)	Chan et al.^66^	Addgene #55788; RRID:Addgene_55788
pcDNA SF-HERC2 F6 (aa 4421–4834)	Chan et al.^66^	Addgene 55789; RRID:Addgene_55789
pcDNA5/FRT/TO: SF-HERC2 (ShB-R)	Chan et al.^66^	Addgene #55613; RRID:Addgene_55613
pcDNA5/FRT/TO: SF-HERC2 C4762S (ShB-R)	Chan et al.^66^	Addgene #55614; RRID:Addgene_55614
pcDNA5/FRT/TO: SF-HERC2 (resistant to HERC2-siRNA #2)	This paper	Internal ID: pYY309
pcDNA5/FRT/TO: SF-HERC2(C4762S) (resistant to HERC2-siRNA #2)	This paper	Internal ID: pYY310
pcDNA5/FRT/TO: SF-HERC2ΔRLD3 (lacking aa 3953–4321)	This paper	Internal ID: pYY323
pcDNA5/FRT/TO: SF-HERC2(E3994A)	This paper	Internal ID: pYY431
pcDNA5/FRTΔTO	This paper	Internal ID: pYY308
pcDNA5/FRTΔTO: ZNRD2 (resistant to ZNRD2-siRNA #2)	This paper	Internal ID: pYY313
pcDNA5/FRTΔTO: ZNRD2(K82A)	This paper	Internal ID: pYY415
pcDNA5/FRTΔTO: ZNRD2(L42E)	This paper	Internal ID: pYY417
pcDNA5/FRTΔTO: ZNRD2-TEV-3xFLAG	This paper	Internal ID: pYY314
pcDNA5/FRTΔTO: ZNRD2(L42E)-TEV-3xFLAG	This paper	Internal ID: pYY377
pcDNA5/FRTΔTO: ZNRD2(L43E)-TEV-3xFLAG	This paper	Internal ID: pYY380
pcDNA5/FRTΔTO: ZNRD2(M48E)-TEV-3xFLAG	This paper	Internal ID: pYY382
pcDNA5/FRTΔTO: ZNRD2(I59E)-TEV-3xFLAG	This paper	Internal ID: pYY367
pcDNA5/FRTΔTO: ZNRD2(D79A)-TEV-3xFLAG	This paper	Internal ID: pYY348
pcDNA5/FRTΔTO: ZNRD2(K82A)-TEV-3xFLAG	This paper	Internal ID: pYY349
pcDNA5/FRTΔTO: ZNRD2(D83A)-TEV-3xFLAG	This paper	Internal ID: pYY350
pcDNA5/FRTΔTO: 3xFLAG-TEV-PhLP1	This paper	Internal ID: pYY277
pcDNA5CMVd3/FRTΔTO: ZNRD2	This paper	Internal ID: pYY421
pcDNA5CMVd3/FRTΔTO: ZNRD2(K82A)	This paper	Internal ID: pYY424
pcDNA5CMVd3/FRTΔTO: ZNRD2(L42E)	This paper	Internal ID: pYY427
pSpCas9(BB)-2A-Puro (pX459)	Ran et al.^67^	Addgene #48139; RRID:Addgene_48139
pX459: hHERC2-gRNA	This paper	Internal ID: pYY327
pX459: hZNRD2-gRNA	This paper	Internal ID: pYY297

**Software and algorithms**		

FlowJo (ver. 10.8.1)	Becton, Dickinson and Company	RRID:SCR_008520; https://www.flowjo.com/solutions/flowjo
Fiji	Schindelin et al.^68^	RRID:SCR_002285; https://fiji.sc/
Image Lab 6.1	Bio-Rad	https://www.bio-rad.com/en-uk/product/image-lab-software?ID=KRE6P5E8Z
MaxQuant software (ver.1.6.17.0)	Cox and Mann^69^	RRID:SCR_014485; https://maxquant.net/maxquant/
Perseus software (ver. 1.6.15.0)	Tyanova et al.^70^	RRID:SCR_015753; https://maxquant.net/perseus/
GraphPad Prism (ver. 9.4.1)	GraphPad Software	RRID:SCR_002798; http://www.graphpad.com/
R (ver. 4.2.0)	R Foundation for Statistical Computing	RRID:SCR_001905; http://www.r-project.org/
AlphaFold2	Jumper et al.^71^	N/A
AlphaFold-Multimer	Evans et al.^35^	N/A
ColabFold (ver. 1.2)	Mirdita et al.^72^	N/A
UCSF ChimeraX (ver. 1.3)	Pettersen et al.^73^	RRID:SCR_015872; https://www.cgl.ucsf.edu/chimerax/
ConSurf Web Server	Ashkenazy et al.^74^	https://consurf.tau.ac.il
Adobe Photoshop	Adobe	RRID:SCR_014199; https://www.adobe.com/products/photoshop.html
Adobe Illustrator	Adobe	RRID:SCR_010279; http://www.adobe.com/products/illustrator.html

**Other**		

DMEM, high glucose, GlutaMAX Supplement, pyruvate	Thermo Fisher Scientific	Cat#10569010
Fetal Bovine Serum	Thermo Fisher Scientific	Cat#10270106
Tetracycline-free Fetal Bovine Serum	BIOSERA	Cat#FB-1001T/500
Expi293 Expression Medium	Thermo Fisher Scientific	Cat#A1435101
GlutaMAX Supplement	Thermo Fisher Scientific	Cat#35050061
DMEM, high glucose, without L-methionine, L-cystine and L-glutamine	Sigma-Aldrich	Cat#D0422
TransIT-293 Transfection Reagent	Mirus	Cat#MIR 2700
Lipofectamine RNAiMAX Transfection Reagent	Thermo Fisher Scientific	Cat#13778150
Rabbit Reticulocyte Lysate Mix	Sharma et al.^75^	N/A
Strep-Tactin Sepharose High-Performance	GE Healthcare	Cat#28-9355-99
Ni-NTA agarose	QIAGEN	Cat#30210
CaptivA Protein A resin	Repligen	Cat#CA-PRI-0100
Pierce ECL Western Blotting Substrate	Thermo Fisher Scientific	Cat#32209
SuperSignal West Pico PLUS Chemiluminescent Substrate	Thermo Fisher Scientific	Cat#34080
Immobilon Western Chemiluminescent HRP Substrate	Millipore	Cat#WBKLS0500
4x Native PAGE Sample Buffer	Thermo Fisher Scientific	Cat#BN2003
NativePAGE 4 to 16%, Bis-Tris Gels	Thermo Fisher Scientific	Cat#BN1002BOX
NativePAGE Running Buffer Kit	Thermo Fisher Scientific	Cat#BN2007

### Resource availability

#### Lead contact

Further information and requests for resources and reagents should be directed to and will be fulfilled by the lead contact, Ramanujan Hegde (rhegde@mrc-lmb.cam.ac.uk).

#### Materials availability

All unique/stable materials generated in this study are available by request from the lead contact.

### Experimental model and study participant details

#### Cell culture

HEK293T and MCF7 cells were cultured in Dulbecco’s Modified Eagle’s Medium (DMEM) supplemented with 10% fetal bovine serum (FBS). Flp-In T-REx 293 cells were maintained in DMEM with 10% FBS, 15 μg/ml blasticidin S, and 100 μg/ml Zeocin. Flp-In T-REx 293-derived knockout and stable cell lines were generated as described in the “method details” section and maintained in the respective selection media. For stable Flp-In T-REx 293 cell lines containing doxycycline-inducible expression constructs, tetracycline-free FBS was used instead of standard FBS. All the Flp-In T-REx 293-derived cell lines were grown in the absence of antibiotics for at least two passages prior to use in assays. Expi293F suspension cells were grown in Expi293 Expression Medium (Thermo Fisher Scientific). Plasmid DNA transfections for generating knockout and stable cell lines were carried out using TransIT-293 transfection reagent (Mirus) according to the manufacturers’ instructions. All the other plasmid DNA transfections were performed using PEI MAX (Polysciences) with a DNA:PEI (w/w) ratio of 1:4 for adherent cells and 1:3 for suspension cells. For siRNA-mediated knockdown, cells were transfected with siRNAs using Lipofectamine RNAiMAX (Thermo Fisher Scientific) according to the manufacturers’ instructions. Cell lines were routinely checked for mycoplasma contamination and verified to be negative. Identities of the cell lines were verified by antibiotic resistance markers and/or by western blotting for the product of knockout alleles. All cell lines used in this study are listed in the key resources table.

### Method details

#### Constructs

All plasmids used in this study were constructed by PCR-based cloning methods and verified by sequencing. The cDNAs encoding the following human proteins with their respective NCBI RefSeq accession numbers were obtained by reverse-transcription PCR using first-strand cDNA prepared from HEK293T cells: CCT1 (NP_110379.2), CCT2 (NP_006422.1), CCT3 (NP_005989.3), CCT4 (NP_006421.2), CCT5 (NP_036205.1), CCT6A (NP_001753.1), CCT7 (NP_006420.1), CCT8 (NP_006576.2), ZNRD2 (NP_006387.1), NCOA4 (NP_001138734.1), and PhLP1 (NP_005379.3).

Constructs for *in vitro* translation in rabbit reticulocyte lysate (RRL) were based on a pSP64 vector containing a C-terminal TwinStrep tag (TST) or 3xFLAG tag. The constructs for *in vitro* translation in the PURE system^32^ were based on the PURExpress control DHFR plasmid provided in the PURExpress *In Vitro* Protein Synthesis Kit (New England Biolabs). To express 3xFLAG-tagged CCT subunits in mammalian cells, the coding sequences of individual subunits were subcloned into a pcDNA5/FRT/TO vector (Thermo Fisher Scientific) containing a C-terminal 3xFLAG tag. The dual color fluorescent reporter constructs were based on a pcDNA5/FRT/TO vector containing a GFP-P2A-RFP cassette (GFP and RFP separated by the P2A sequence), with the coding sequence of a protein of interest being inserted upstream of and in-frame with the GFP sequence. Monomeric EGFP and mCherry were used, but are referred to as GFP and RFP for simplicity. A previously described pcDNA5/FRT/TO-based GFP-P2A-RFP construct without any test substrate was used as a control reporter.^65^ The mutants of CCT4 were generated by site-directed mutagenesis using CCT4-TST in pSP64 and subsequently subcloned into the pcDNA5/FRT/TO-based dual color fluorescent reporter plasmid.

The plasmids encoding full-length HERC2 (WT and C4762S mutant) as well as HERC2 fragments were obtained from Addgene (#55613,^66^ #55614,^66^ #55784,^66^ #55785,^66^ #55786,^66^ #55787,^66^ #55788,^66^ #55789^66^). The full-length HERC2 constructs were modified to confer resistance to HERC2 siRNA #2, and these siRNA-resistant versions were used throughout the study. The HERC2 construct lacking RLD3 domain (amino-acid residues 3953–4321) and the one carrying the E3994A mutation were generated from the siRNA-resistant WT construct. The ZNRD2 and PhLP1 constructs were based on pcDNA5/FRT/TO-derived vectors lacking the Tet operator (referred to as pcDNA5/FRTΔTO). The coding sequence of ZNRD2 was modified to confer resistance to ZNRD2 siRNA #2 and cloned into a pcDNA5/FRTΔTO vector with or without a C-terminal 3xFLAG tag, whereas the coding sequence of PhLP1 was cloned into a pcDNA5/FRTΔTO vector carrying an N-terminal 3xFLAG tag. The single-point mutants of ZNRD2 were generated using site-directed mutagenesis. Subsequently, the plasmids encoding untagged ZNRD2 and its mutants containing L42E or K83A mutation were further modified to express the corresponding ZNRD2 gene under the control of a weakened CMV promoter (CMVd3 promoter^76^). These pcDNA5CMVd3/FRTΔTO-based plasmids were used to generate the cell lines used in cell growth competition experiments.

All the CCT subunits and ZNRD2 constructs with a 3xFLAG tag contain a TEV cleavage site between the gene of interest and the 3xFLAG tag, and all the HERC2 constructs contain N-terminal 3xFLAG tag and Strep-tag II (referred to as SF-tag by the depositing lab). However, these constructs are described simply as 3xFLAG-tagged constructs in figures and text.

#### Generation of knockout cell lines

HERC2-KO and ZNRD2-KO Flp-In T-REx 293 cells were generated by CRISPR/Cas9-mediated gene disruption, essentially as described previously.^67^ The guide RNAs (gRNAs) targeting exon 10 of human *HERC2* (5’-AAGCTCGTTGTCTTGTGGAA-3’) and exon 2 of human *ZNRD2* (5’-ATGGGCGACTATCTGCTGCG-3’) were cloned into the pX459 plasmid.^67^ Parental Flp-In T-REx 293 cells were transfected with the resulting pX459 plasmid encoding the appropriate gRNA. After 24 h, transfected cells were selected for 48 h with 2 μg/ml puromycin, followed by an additional 48 h incubation in the absence of puromycin. Cells were then plated into 96-well plates at a density of 0.5 cells/well to isolate single-cell clones. After expansion, clones were screened for successful knockout by western blotting. During the isolation and screening of single-cell clones, cells were maintained in media containing 15 μg/ml blasticidin S and 100 μg/ml Zeocin. For each gRNA, multiple clones were established, and the phenotypes were verified to be the same among them using fluorescent reporter assays. One representative knockout clone per gRNA was used throughout the present study.

#### Generation of stable cell lines

Stable Flp-In T-REx 293 cell lines containing a doxycycline-inducible 3xFLAG-HERC2 construct or its C4762S mutant were generated using the Flp-In system (Thermo Fisher Scientific) according to the manufacturer’s instructions. Briefly, Flp-In T-REx 293 cells were co-transfected with pOG44 and the appropriate pcDNA5/FRT/TO-based HERC2 construct. After 48 h, cells were split into media containing 100 μg/ml hygromycin B to select for cells with a targeted integration of the expression vector into the unique FRT site as well as 15 μg/ml blasticidin S to maintain the expression of the Tet repressor. After expansion, a pooled population of resistant cells was assessed for the expression of each construct and used in assays. ZNRD2-KO Flp-In T-REx 293 cell lines stably and constitutively expressing untagged or 3xFLAG-tagged ZNRD2 constructs were likewise established using the Flp-In system in combination with the appropriate pcDNA5/FRTΔTO- or pcDNACMVd3/FRT/ΔTO-based plasmids, which allow constitutive (i.e., doxycycline-independent) expression of the construct in Flp-In T-REx 293-derived cells. ZNRD2-KO rescue cell lines used in Figures 6D and S4D and were generated with pcDNA5/FRTΔTO-based plasmids, while those used in Figure 7 (cell growth competition assays) were with pcDNACMVd3/FRT/ΔTO-based plasmids. ZNRD2-KO cells stably integrated with an empty pcDNA5/FRTΔTO vector were also generated and used as a control cell line. These ZNRD2-KO-derived stable cell lines were polyclonal and used in assays without further single-cell cloning.

#### Recombinant proteins

Recombinant human full length HERC2, HERC2(C4762S), HERC2ΔRLD3, and ZNRD2 were purified from Expi293F cells via the 3xFLAG tag on each protein. Cells were transfected at a density of 2.5–3.5 x 10^6^ cells/ml with 1 μg of plasmid DNA per ml of culture using PEI MAX. The following day, cells were harvested and either immediately lysed or snap-frozen and stored −80°C until lysis. Cells were lysed by passing through a 23G needle or douncing in lysis buffer [50 mM HEPES-KOH, pH 7.4, 100 mM KOAc, 5 mM Mg(OAc)_2_, 0.01% digitonin, 1 mM DTT, and 1x protease inhibitor cocktail (EDTA-free cOmplete from Roche)]. Lysates were centrifuged at 15,000 rpm in a tabletop centrifuge at 4°C for 10–15 min, and the supernatant was passed over a column of packed anti-FLAG M2 affinity gel (Sigma-Aldrich). Columns were washed with 30–35 column volumes each of lysis buffer, high salt buffer (50 mM HEPES-KOH, pH 7.4, 400 mM KOAc, 5 mM Mg(OAc)_2_, 0.01% digitonin, 1 mM DTT) and physiological salt buffer (PSB: 50 mM HEPES-KOH, pH7.4, 100 mM KOAc, 5 mM Mg(OAc)_2_). Proteins were eluted three times with 0.2 mg/ml 3xFLAG peptide (Sigma-Aldrich) in PSB, with a 20-min incubation each at room temperature. Human His-Ubiquitin (WT and K0), GST-UBE1, and UBCH5a used for *in vitro* ubiquitination reactions were purchased from Boston Biochem.

#### Flow cytometry analysis

Dual color fluorescent reporter constructs were transiently transfected into cells grown in 12-well plates using PEI MAX. Where indicated, HERC2 and ZNRD2 constructs were co-transfected. The amounts of each construct used for transfection were as follows: fluorescent reporter constructs, 100 ng/well; HERC2 constructs, 100 ng/well; and ZNRD2 constructs, 100 ng (Figure 3) or 10 ng (Figures 5 and S7). The total amount of transfected DNA was maintained constant at 500 ng/well by adding empty pcDNA5/FRT/TO vector. In cases where Flp-In T-REx 293 and its derivative cell lines were employed, expression of the reporter and HERC2 constructs was induced with 1 μg/ml doxycycline, which was added to wells prior to transfection. At 18–22 h post-transfection, cells were washed once with phosphate-buffered saline (PBS), trypsinized, and harvested in ice-cold PBS containing 2% FBS. Cells were then pelleted by centrifugation and resuspended in ice-cold PBS containing 2% FBS, followed by passing through a 70-μm cell strainer to remove any clumped cells. Data were collected using a Beckton Dickinson LSRFortessa flow cytometer and analyzed using FlowJo software. Each experiment was internally controlled, and 20,000–30,000 transfected cells were analyzed per condition. The histograms within any experiment (graph) can be directly compared, whereas those across experiments (graphs) cannot because fluorescence intensity values depend on the cytometer settings and calibration. In general, however, the cytometer was set such that untagged GFP and RFP expressed from the control GFP-P2A-RFP construct generate roughly equal fluorescence intensity values that fall on a diagonal line across a wide range of intensities (Figure 1A, black dots).

For experiments using knockdown cells, cells were reverse-transfected in 6-well plates with the indicated siRNAs using Lipofectamine RNAiMAX. Two days after siRNA transfection, cells were re-plated into 12-well plates and grown for another day before being transfected with fluorescent reporter and other DNA constructs. In the experiment shown in Figure S3A, two rounds of siRNA transfection were conducted as follows. Cells were reverse-transfected with siRNAs in 6-well plates and incubated for 2 days before a second round of siRNA transfection using a forward transfection method. Three days after the initial siRNA transfection, cells were re-plated into 12-well plates and were transfected the following day with the fluorescent CCT4 reporter. Unless specified otherwise, HERC2 siRNA #2 and ZNRD2 siRNA #2 were used for fluorescent reporter assays in knockdown cells. Data collection and analysis were carried out as described above.

Where immunoblotting analysis of cells was desired, an aliquot of cells was harvested and washed once in ice-cold PBS before being lysed in SDS lysis buffer (1% SDS/100 mM Tris-HCl, pH 8.0). Lysates were heat-denatured at 95°C for ∼10 min with occasional vortex mixing to shear genomic DNA.

#### Analysis of steady-state levels of endogenous CCT subunits after CCT2 knockdown

To analyze steady-state levels of endogenous CCT subunits in CCT2 knockdown cells, HEK293T cells were reverse-transfected in 12-well plates with CCT2-targeting siRNAs and incubated for 72 h. Cells were washed once with PBS, lysed in SDS lysis buffer, and heat-denatured as described in the previous section. For co-depletion of CCT2 and HERC2, cells were reverse-transfected in 12-well plates with the indicated pairs of siRNAs at a 1:1 ratio for 72 h and then lysed as above. To assess the effects of ZNRD2 mutations on degradation of endogenous orphan CCT subunits, CCT2 siRNA #2 and ZNRD2 siRNA #2 were co-transfected at a 1:1 ratio into cells in 6-well plates by reverse transfection. The following day, cells were transfected with 10 ng of the indicated ZNRD2 constructs or empty vector (as a control), 100 ng of GFP, and 890 ng of empty vector. Approximately 72 h after the initial siRNA transfection, GFP-positive (i.e., transfection-positive) cells were FACS-sorted and lysed as above.

#### Mammalian *in vitro* translation

SP6 polymerase-driven *in vitro* transcription was performed as previously described,^75^ using PCR products as the template. Transcription reactions were conducted at 37°C for 1 h with 5–10 ng/μl purified PCR product and 0.4 U/μl SP6 polymerase (Promega) in 40 mM HEPES, pH 7.4, 6 mM MgCl_2_, 20 mM spermidine, 10 mM reduced glutathione, 0.5 mM ATP, 0.5 mM UTP, 0.5 mM CTP, 0.1 mM GTP, 0.5 mM CAP, and 0.4–0.8 U/μl RNAsin (Promega). The transcription reactions were directly used for *in vitro* translation without further purification. *In vitro* translation in rabbit reticulocyte lysate (RRL) was performed as described previously in detail.^75^^,^^77^ In brief, crude reticulocyte lysate (Green Hectares) was pre-treated with micrococcal nuclease to digest endogenous mRNAs. Translation reactions typically contained 33% by volume nuclease-treated RRL, 20 mM HEPES, 10 mM KOH, 50 mM KOAc, 2 mM MgCl_2_, 20 μg/ml pig liver tRNA, 40 μg/ml creatine kinase, 12 mM creatine phosphate, 1 mM ATP, 1 mM GTP, 1 mM reduced glutathione, 0.3 mM spermidine, 40 μM of each amino acid except methionine, and either 40 μM unlabelled methionine or 0.5 μCi/μl ^35^S-methionine. Each translation reaction was programmed with the transcription reaction, which comprised 5% of the total translation reaction volume, and was performed at 32°C for 30–45 min. In experiments where affinity-purification of ubiquitinated products was desired, the translation reactions were supplemented with 10 μM His-Ubiquitin and incubated at 32°C for 60 min. Immediately after translation, samples were placed on ice and analyzed directly or subjected to further manipulations as described below.

#### Affinity-purification from RRL translation reactions

Affinity-purifications of CCT substrates under native conditions were done at 0–4°C as follows. For identification of quality control factors associated with CCT subunits, 600 μl translation reactions were directly incubated with 15 μl of StrepTactin High-Performance Sepharose at 4°C for 1.5 h with end-over-end rotation. The resin was washed 5 times with physiological salt buffer-125 (PSB125: 50 mM HEPES-KOH, 125 mM KOAc, 2 mM Mg(OAc)_2_), transferred to a new tube, and subjected to mass spectrometry as described below. For validation of key protein-protein interactions and analysis of interaction of various CCT4 mutants with ZNRD2, 200-μl translation reactions were likewise incubated with 10 μl of StrepTactin High-Performance Sepharose at 4°C for 1.5–2 h. After washing 5 times with PSB125, the resin was transferred to a new tube, followed by protein elution with SDS sample buffer. To recover ubiquitinated products from translation reactions, the reactions (typically 12–15 μl) were done in the presence of His-Ubiquitin as described above. A small aliquot of the reaction was set aside for analysis of total translation products, and the remainder (typically ∼10 μl) was adjusted to 100 mM Tris-HCl, pH 8.0 and 1% SDS in a final volume of 100 μl and denatured at 95°C for 5–10 min. The denatured samples were cooled, diluted 10-fold in His-PD buffer (1xPBS supplemented with 250 mM NaCl, 0.5% Triton X-100, and 20 mM imidazole), and then mixed with 10 μl of Ni-NTA agarose (Qiagen) at 4°C for 1.5 h with end-over-end rotation. The resin was washed 3 times with His-PD buffer, and samples were eluted with SDS sample buffer containing 50 mM EDTA.

#### Label-free quantitative mass spectrometry

*In vitro* translation and affinity-purification were performed as described above. TwinStrep-tagged CCT subunit constructs (CCT1–8) served as bait proteins to identify interaction partners, whereas 3xFLAG-tagged CCT3 and CCT7 served as negative controls. In addition, mock translation reaction was used as another negative control. The experiment was done in triplicate for each condition. Affinity-purified protein samples on beads were resuspended in 50 μl of 20 mM HEPES, followed by reduction in DTT at 56°C and alkylation with iodoacetamide in the dark at room temperature. Samples were then digested with 200 ng of trypsin (Promega) overnight at 37°C. After centrifugation at 10,000 x g for 5 minutes, the supernatant was transferred to a new tube. The beads were washed once with 30 μl of 5% formic acid (FA), and the solution was combined with the corresponding supernatant. The resulting peptide mixtures were desalted using a home-made C18 (3M Empore) stage tip that contained 2 μl of Poros Oligo R3 resin (Thermo Fisher Scientific). Bound peptides were eluted from the stage tip with 30–80% acetonitrile (MeCN) and partially dried in a SpeedVac (Savant).

Peptides were separated on an Ultimate 3000 RSLC nano System (Thermo Scientific) fitted with a 75 μm x 25 cm nanoEase C18 T3 column (Waters), using mobile phases buffer A (2% MeCN, 0.1% FA) and buffer B (80% MeCN, 0.1% FA). Eluted peptides were introduced directly via a nanospray ion source into a Q Exactive Plus hybrid quadrupole-Orbitrap mass spectrometer (Thermo Fisher Scientific). The mass spectrometer was operated in data dependent mode. MS1 spectra were acquired from 380–1600 m/z at a resolution of 70K, followed by MS2 acquisitions of the 15 most intense ions with a resolution of 17.5K and NCE of 27%. MS target values of 1e^6^ and MS2 target values of 5e^4^ were used. Dynamic exclusion was set for 40 sec.

The acquired raw data files were processed for protein identification and quantification with MaxQuant software (version 1.6.17.0)^69^ employing the Andromeda search engine.^78^ The data were searched against the *Homo sapiens* reviewed UniProt FASTA database (Dec 2020). Carbamidomethylation of cysteine was set as fixed modification, while oxidation of methionine and protein N-terminal acetylation were set as variable modifications. Up to two missed cleavage sites of trypsin were allowed. Protein quantification was performed using the label-free quantitation (LFQ) algorithm in MaxQuant, and MaxQuant output was further processed with Perseus software (version 1.6.15.0).^70^ Briefly, potential contaminants, reverse hits, hits only identified by site, and hits with only 1 unique and razor peptide were filtered out prior to log_2_ transformation of the LFQ intensities. Replicate samples were grouped, and the data was filtered to retain proteins with three valid values in at least one group. Missing values were then imputed from a normal distribution with a width of 0.3 and a downshift of 1.8.

For statistical analysis, three different negative controls (mock translation, CCT3-3xFLAG, and CCT7-3xFLAG) were grouped and used as a common control for all the test pull-downs. To identify specific interactors of each bait protein, statistical analysis was performed using a two-tailed Student’s *t*-test with Benjamini-Hochberg correction for multiple comparisons. Volcano plots were generated using R (version 4.2.0). Given the use of the RRL-based *in vitro* translation system, a similar analysis was also conducted by searching the mass spectra against the *Oryctolagus cuniculus* UniProt FASTA database (Feb 2021). Although similar results were obtained, ZNRD2 was not identified, most likely due to incomplete annotation of the *O. cuniculus* genome. The results presented in this study were based on the analysis with the *H. sapiens* database.

#### Co-immunoprecipitation in cells

To analyze interactions between 3xFLAG-tagged CCT subunits and endogenous proteins, HEK293T cells grown in 6-well plates were transiently transfected with 1 μg of CCT-3xFLAG constructs using PEI MAX. Where knockdown of HERC2 and ZNRD2 was desired, HEK2933T cells were reverse-transfected with siRNAs against HERC2 (#2) and ZNRD2 (#2) in 6-well plates using Lipofectamine RNAiMAX. After 2 days, cells were re-plated into 6-well plates and cultured for one day before transfection with DNA constructs. Two days after plasmid DNA transfection, cells were harvested and lysed on ice in native IP buffer (50 mM HEPES-KOH, 125 mM KOAc, 2 mM Mg(OAc)_2_, 1 mM DTT, 0.01% digitonin) supplemented with 1x protease inhibitor cocktail. Lysates were spun at 15,000 rpm for 10 min at 4°C using a tabletop centrifuge, and the supernatants (cytosolic fractions) were incubated with 10 μl of anti-FLAG M2 affinity gel at 4°C for 1.5–2 h with end-over-end rotation. The resin was then washed 4 times with native IP buffer, and bound proteins were eluted with SDS sample buffer. Analysis of interaction between 3xFLAG-HERC2 constructs and endogenous ZNRD2 was performed in the same manner. For analysis of interactions between ZNRD2 mutants and endogenous proteins (Figure 5D), ZNRD2-KO cells grown in 6-cm dishes were transiently transfected with 2 μg of ZNRD2-3xFLAG constructs using PEI MAX. Lysate preparation and immunoprecipitation with anti-FLAG M2 affinity gel were done as described above. After washing the resin 4 times with native IP buffer, proteins were eluted with 0.25 mg/mL 3xFLAG peptide in NT buffer (50 mM HEPES-KOH, 125 mM NaCl, 2 mM Mg(OAc)_2_, 1 mM DTT, 0.1% TritonX-100) for 30 min at room temperature with constant gentle mixing. To analyze interactions between endogenous CCT subunits and overexpressed 3xFLAG-tagged ZNRD2 constructs or PhLP1 (Figure 6C), HEK293T cells grown in 10-cm plates were transiently transfected with 5 μg of ZNRD2-3xFLAG (WT or L42E) or 3xFLAG-PhLP1. Cells were subjected to anti-FLAG immunoprecipitation as described above, using 20 μl of anti-FLAG affinity gel. Protein elution was conducted at room temperature using 0.25 mg/mL 3xFLAG peptide in native IP buffer for 30 min with constant gentle mixing. For sequential co-immunoprecipitation assays, one confluent 15-cm dish of ZNRD2-KO cells stably overexpressing ZNRD2-3xFLAG was subjected to anti-FLAG immunoprecipitation as described above, using 20 μl of anti-FLAG M2 affinity gel. ZNRD2-KO cells stably overexpressing untagged ZNRD2 were also used as negative control in the anti-FLAG immunoprecipitation. After incubation and washing, protein elution was performed at room temperature for 30 min using 0.25 mg/mL 3xFLAG peptide in native IP with constant gentle mixing. A 10% portion of the immunoprecipitated fraction was set aside for analysis, and the remainder was divided into two equal aliquots and subjected to the second immunoprecipitation as follows. Each aliquot was diluted ∼5-fold in native IP buffer and incubated at 4°C with 4 μg of either rabbit control IgG or anti-CCT4 antibody (Bethyl, Cat# A304-725A) for 1.5 h and subsequently with 10 μl of protein A resin (Repligen) for another 1.5 h with end-over-end rotation. The resin was washed 4 times with native IP buffer, and proteins were eluted with SDS sample buffer.

#### Pulse-chase experiments

MCF7 cells were transfected with control siRNA or HERC2 siRNA #2 using Lipofectamine RNAiMAX for 96 h. Cells were depleted of free cold methionine by incubation in methionine-free DMEM (Sigma-Aldrich) for 30 min. Cells were then labelled with ^35^S-methionine at 100 μCi/ml for 30 min, washed in PBS, and either collected immediately or chased for 4 h in complete medium. Cells were lysed in SDS lysis buffer. After heating to 95°C for 5 min, lysates were diluted 10-fold in IP buffer (50 mM HEPES, 150 mM NaCl, 1% Triton X-100) and centrifuged for 10 min at 15000 rpm to remove insoluble material. Supernatants were incubated with rabbit anti-CCT7 antibody (Bethyl) at 1:500 dilution for 1 h at 4°C, and a further 1-2 h at 4°C with 7.5 μl protein A resin (Repligen). Beads were washed 3 times in IP buffer and eluted in SDS sample buffer for analysis by SDS-PAGE and autoradiography. Densitometry was performed with Fiji software. CCT7 intensity values for each sample were normalized according to the intensity of the corresponding input lysate. Data plotting and statistical analysis were performed using GraphPad Prism (ver. 9.4.1).

#### PURE *in vitro* translation

Translations in the PURE system (Protein synthesis Using Recombinant Elements) were performed as described previously, using reagents prepared in-house^79^^,^^80^ or the PURExpress Δ (aa, tRNA) Kit (New England Biolabs). Translation reactions were assembled with 10 ng/μl of the appropriate plasmid DNA (encoding CCT4-3xFLAG or each of untagged CCT subunits) and 1 μCi/μl ^35^S-methionine. After translation at 37°C for 1 h, samples were layered onto a 200 μl of 5–25% sucrose gradient directly or after dilution in PSB. The gradients were prepared in 7 x 20 mm centrifuge tubes (Beckman Coulter, Cat# 343775) by successively layering 40 μl each of 25%, 20%, 15%, 10% and 5% sucrose in PSB and allowing to equilibrate for 30–60 min at 4°C. Samples were centrifuged in a TLS-55 rotor (with suitable tube adaptors; Beckman Coulter) at 55,000 rpm at 4°C with slow acceleration and deceleration for 2 h 25 min. Following centrifugation, eleven 20-μl fractions were collected manually from the top and analyzed by SDS-PAGE and autoradiography. Fractions containing soluble CCT subunits were combined and used for *in vitro* ubiquitination assays.

#### *In vitro* ubiquitination reactions

To detect E3 ligase activities associated with nascent CCT subunits, TwinStrep-tagged CCT subunits were synthesised in RRL, affinity-purified, and subjected to ubiquitination reactions as follows. Translation reactions (typically ∼45 μl) were diluted ∼10-fold in PSB125 and incubated with 15 μL StrepTactin High-Performance Sepharose at 4°C for 1.5 h with end-over-end rotation. After washing 4 times with PSB125, the resin was divided into three equal aliquots. One aliquot was incubated with a ubiquitination reaction mixture (PSB125 containing 1 mM ATP, 10 mM creatine phosphate, 40 μg/ml creatine kinase, 10 μM His-Ubiquitin, 100 nM GST-UBE1, and 250 nM UBCH5a), the second was incubated with the ubiquitination reaction mixture lacking GST-UBE1, and the third was incubated with the ubiquitination reaction mixture supplemented with RRL. Ubiquitination reactions were carried out on beads at 32°C for 30 min and stopped by adjusting to 1% SDS and 0.1 M Tris-HCl, pH8.0 and heating at 95°C. For *in vitro* reconstitution of HERC2- and ZNRD2-mediated ubiquitination of CCT subunits, soluble CCT substrate produced in the PURE system was mixed with 1 mM ATP, 10 mM creatine phosphate, 40 μg/ml creatine kinase, 10 μM His-Ubiquitin, 100 nM GST-UBE1 and 250 nM UBCH5a in PSB, together with or without ∼15 nM 3xFLAG-HERC2 and ∼800 nM ZNRD2-3xFLAG. Where indicated, His-Ubiquitin-K0, 3xFLAG-HERC2(C4762S) and 3xFLAG-HERC2ΔRLD3 mutants were used instead of their WT counterparts. Reactions were incubated at 32°C for 30 min and stopped by denaturation at 95°C in 1% SDS, 0.1 M Tris-HCl, pH8.0. Autoubiquitination assays using recombinant HERC2 proteins were likewise conducted in PSB containing 1 mM ATP, 10 mM creatine phosphate, 40 μg/ml creatine kinase, 10 μM His-Ubiquitin and 250 nM UBCH5a with or without 100 nM GST-UBE1. Ubiquitinated products were recovered by His-Ubiquitin pull-down as described above.

#### SDS-PAGE and immunoblotting

Protein samples were prepared as described above. Where possible and appropriate, protein concentrations of total cell lysates and cytosolic extracts were normalized based on A280 readings. Samples were mixed with SDS sample buffer, separated by Tris-Tricine SDS-PAGE, and electrotransferred to a 0.2 μm nitrocellulose membrane. Blots were blocked in 5% nonfat dry milk in PBS-T (PBS containing 0.1% Tween 20) at room temperature for 1 h and incubated with the appropriate primary antibodies at 4°C overnight. After washing in PBS-T, blots were incubated with HRP-conjugated secondary antibodies for 1–1.5 h at room temperature and then washed extensively in PBS-T. Blots were subsequently developed using Pierce ECL substrate (Thermo Fisher Scientific), SuperSignal West Pico Chemiluminescent Substrate (Thermo Fisher Scientific), or Immobilon Western Chemiluminescent HRP Substrate (Millipore) before being exposed to X-ray films or imaged on a ChemiDoc MP Imaging System (Bio-Rad). Figures were assembled using Image Lab 6.1 software (Bio-Rad), Adobe Photoshop, and Adobe Illustrator.

#### Sucrose gradient fractionation of cytosolic extracts

Cytosolic fractions were prepared from one 10-cm plate of cells as described in the “co-immunoprecipitation in cells” section, using 60–120 μL (typically 100 μL) of buffer for cell lysis to obtain less-diluted cytosolic extracts. For experiments using HERC2-overexpressing cells, stable Flp-In T-REx 293 cells containing doxycycline-inducible 3xFLAG-HERC2, 3xFLAG-HERC2(C4762S), or empty vector were treated with 1 μg/mL doxycycline for 4–5 days (typically 4 days) before being harvested for cytosol extraction. Cells were split once during the doxycycline treatment. For experiments involving HERC2 or ZNRD2 knockdown, HEK293T cells were reverse-transfected with the appropriate siRNAs in 6-cm plates using Lipofectamine RNAiMAX. Two days later, cells were transfected again with the same siRNAs as those used in the first round of siRNA transfection. The following day, cells were split into 10-cm plates, cultured for another two days, and harvested for preparation of cytosolic fractions. Protein concentrations of cytosolic extracts were adjusted based on A280 measurements, and 20 μl of the normalized extract was layered onto a 200 μl of 5–45% sucrose gradient, which had been prepared by overlaying 40 μl each of 45%, 35%, 25%, 15%, and 5% sucrose in PSB125 and allowing to equilibrate for ∼1 h at 4°C. Samples were centrifuged in a TLS-55 rotor (with suitable tube adaptors; Beckman Coulter) at 55,000 rpm for 2 h at 4°C with slow acceleration and deceleration. Following centrifugation, eleven 20-μl fractions were collected manually from the top and analyzed by SDS-PAGE and immunoblotting as described above using antibodies against CCT4 and CCT7. To analyze the distribution of CCT subunits of interest across the gradient, chemiluminescent blots were imaged on a ChemiDoc MP Imaging System (Bio-Rad), and the intensities of unsaturated bands were quantified by densitometry using Image Lab 6.1 software (Bio-Rad). The relative band intensity in each fraction was calculated as a proportion of the total signal intensity across all fractions. The proportion of unassembled CCT subunits migrating in fractions 2–4 was used for statistical analysis shown in Figure 3B. Generation of graphs and statistical analyses were done using GraphPad Prism (ver. 9.4.1).

#### Analysis of cytosolic extracts using native PAGE

Cytosolic extracts of cells overexpressing 3xFLAG-HERC2 (WT or C4762S) were prepared as described in the previous section. For experiments using HERC2-depleted MCF7 cells, MCF7 cells were transfected with control siRNA or HERC2 siRNA #2 for 4 days. Preparation of cytosolic extracts was performed essentially as described above, except that PSB containing 0.01% digitonin and 1x protease inhibitor cocktail was used for cell lysis. After normalization of protein concentrations based on A280 readings, samples were subjected to blue native PAGE using NativePAGE Bis-Tris Gel system (Thermo Fisher Scientific) according to the manufacturer’s instructions. Briefly, the normalized cytosolic extracts were mixed with 4x Native PAGE Sample Buffer (Thermo Fisher Scientific) and loaded on 4–12% NativePAGE Bis-Tris gels (Thermo Fisher Scientific). Electrophoresis was performed at 150 V at room temperature using pre-chilled anode buffer and dark blue cathode buffer. When the dye front migrated one-thirds of the way down the gel, the cathode buffer was switched to light blue cathode buffer. Following electrophoresis, gels were soaked in Tris-Glycine-SDS buffer (25 mM Tris, 192 mM glycine, 0.1 % SDS) for 10 min, and proteins were electrotransferred to a 0.45 μm PVDF membrane. Blots were incubated in 8% acetic acid for 15 min, rinsed with deionized water, air-dried, incubated briefly with methanol, and rinsed again with deionized water. Blocking and immunodetection were as described above.

#### Cell growth competition assay

ZNRD2-KO cells complemented with untagged ZNRD2, ZNRD2(K82A), or ZNRD2(L42E) were used. These stable cell lines contained the respective exogenous ZNRD2 construct at the unique FRT site (i.e., the same site) in the genome. Cells expressing WT ZNRD2 construct were mixed at a 1:1 ratio with cells expressing each of the matched mutants (K82A or L42E) and then co-cultured on 10-cm plates for 4 weeks, with passaging every 2–3 days. Aliquots of the co-cultured cells were harvested, pelleted, and snap-frozen at the start of co-culture and the indicated time points for the downstream analysis. Genomic DNA was extracted and purified from frozen cell pellets using DNeasy Blood & Tissue Kit (QOAGEN), and the exogenous ZNRD2 sequences at the FRT site were specifically amplified by genomic PCR using a pair of primers, gPCR-Z-F (5’-CTGCTTCGCGATGGGCGGTAG-3’) and gPCR-Z-R (5’-TCTAGACTCGAGTTATCAATGCTGCAAC-3’). After purification, amplicons were directly Sanger sequenced using the primer gPCR-Z-F to assess the proportion of WT and mutant ZNRD2 alleles present in each co-culture at each time point.

#### Structural modelling of the CCT4-ZNRD2-HERC2 complex

The structure of human ZNRD2 in complex with human CCT4 and RLD3 domain of human HERC2 (aa residues 3951–4320) was predicted using AlphaFold-Multimer.^35^^,^^71^ The prediction was executed through ColabFold (version 1.2)^72^ running on a local computing cluster using the default settings without AMBER relaxation. Five models were generated, and the top-ranked model based on AlphaFold predicted TM-score was used in the present study. The AlphaFold models of monomeric human CCT4 and ZNRD2 were downloaded from AlphaFold Protein Structure Database.^64^ Structural analysis and figure preparation were done using ChimeraX (version 1.3),^73^ including structural alignments, molecular lipophilicity potential calculation, and Coulombic electrostatic potential calculation. Sequence conservation score was calculated using the ConSurf server (https://consurf.tau.ac.il)^74^ and mapped to the molecular surface of ZNRD2 in the predicted complex model using ChimeraX.

### Quantification and statistical analysis

Densitometric analyses of autoradiographs and immunoblots were performed as described in the relevant method sections. All bar graphs show mean ± SD, with dots indicating individual data points, as indicated in the figure legends. For analysis of endogenous CCT7 degradation by pulse-chase assays (Figure 2E), statistical analysis was done in GraphPad Prism using a two-tailed Student’s *t*-test to compare HERC2 knockdown and control knockdown conditions. The *p*-value was indicated in the figure and the figure legend. For quantification of the proportion of unassembled CCT4 and CCT7 by sucrose gradient fractionation (Figure 3B), statistical analysis was performed in GraphPad Prism using one-way ANOVA with Dunn’s multiple comparisons test as indicated in the figure legend. Statistical significance is reported in the figure with the following symbols: ^∗∗^*p*<0.01 and ^∗∗∗^*p*<0.001. In these densitometry-based analyses, *p*-values of <0.05 were considered to be statistically significant. Mass spectrometry data was analyzed using MaxQuant and Perseus as described above. To identify specific interactors of each bait protein, statistical analysis was performed in Perseus using a two-tailed Student’s *t*-test with Benjamini-Hochberg correction for multiple comparisons. The processed data used to generate the volcano plots is available in Table S1.

## Data Availability

• The mass spectrometry proteomics data have been deposited to the ProteomeXchange Consortium via the PRIDE partner repository with the dataset identifier PXD042773. The processed proteomics data is available in this paper’s supplemental information. Previously published structural models are available from the PDB (accession numbers 7LUM, 6HCZ, and 3KCI) and the AlphaFold protein structure database (accession numbers AF-P50991-F1-model_v2 and AF-O60232-F1-model_v2). • This paper does not report original code. • Any additional information required to reanalyze the data reported in this paper is available from the lead contact upon request.

## Supplemental information

Table S1. Mass spectrometry data analysis, related to Figures 1D and S2
